# Four Novel Phenanthrene Derivatives with *α*-Glucosidase Inhibitory Activity from *Gastrochilus bellinus*

**DOI:** 10.3390/molecules26020418

**Published:** 2021-01-14

**Authors:** Htoo Tint San, Nutputsorn Chatsumpun, Thaweesak Juengwatanatrakul, Natapol Pornputtapong, Kittisak Likhitwitayawuid, Boonchoo Sritularak

**Affiliations:** 1 Department of Pharmacognosy and Pharmaceutical Botany, Faculty of Pharmaceutical Sciences, Chulalongkorn University, Bangkok 10330, Thailand; htootintsan@mohs.edu.mm (H.T.S.); Kittisak.L@chula.ac.th (K.L.); 2 Department of Pharmacognosy, University of Pharmacy, Yangon 11031, Myanmar; 3 Department of Pharmacognosy, Faculty of Pharmacy, Mahidol University, Bangkok 10400, Thailand; nutputsorn.cha@mahidol.ac.th; 4 Faculty of Pharmaceutical Sciences, Ubon Ratchathani University, Ubon Ratchathani 34190, Thailand; thaweesak.j@ubu.ac.th; 5 Department of Biochemistry and Microbiology, Faculty of Pharmaceutical Sciences, Chulalongkorn University, Bangkok 10330, Thailand; Natapol.P@chula.ac.th; 6 Natural Products for Ageing and Chronic Diseases Research Unit, Faculty of Pharmaceutical Sciences, Chulalongkorn University, Bangkok 10330, Thailand

**Keywords:** *Gastrochilus bellinus*, Orchidaceae, gastrobellinol, *α*-glucosidase inhibition, phenanthrene derivatives

## Abstract

Four new phenanthrene derivatives, gastrobellinols A-D (**1–4**), were isolated from the methanolic extract of *Gastrochilus bellinus* (Rchb.f.) Kuntze, along with eleven known phenolic compounds including agrostophyllin (**5**), agrostophyllidin (**6**), coniferyl aldehyde (**7**), 4-hydroxybenzaldehyde (**8**), agrostophyllone (**9**), gigantol (**10**), 4-(methoxylmethyl)phenol (**11**), syringaldehyde (**12**), 1-(4′-hydroxybenzyl)-imbricartin (**13**), 6-methoxycoelonin (**14**), and imbricatin (**15**). Their structures were determined by spectroscopic methods. Each isolate was evaluated for α-glucosidase inhibitory activity. Compounds **1**, **2**, **3**, **7**, **9**, **13**, and **15** showed higher activity than the drug acarbose. Gastrobellinol C (**3**) exhibited the strongest *α*-glucosidase inhibition with an IC_50_ value of 45.92 μM. A kinetic study of **3** showed competitive inhibition on the *α*-glucosidase enzyme. This is the first report on the phytochemical constituents and *α*-glucosidase inhibitory activity of *G. bellinus*.

## 1. Introduction

**Figure 1:**
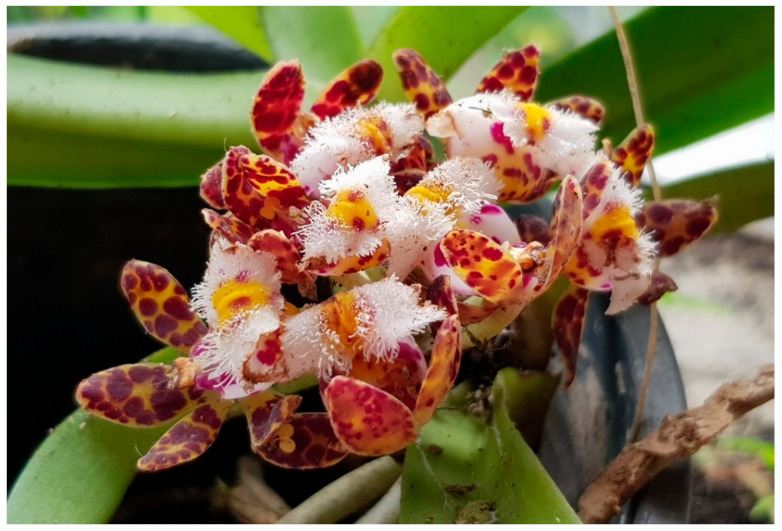
*Gastrochilus bellinus* (Rchb.f.) Kuntze.

Diabetes mellitus (DM), one of the most common metabolic diseases, is characterized by high blood glucose levels due to a deficiency of insulin in the body or insufficient insulin sensitivity [1]. *α*-Glucosidase inhibitors (AGIs), for example, acarbose and voglibose, are the drugs of choice for treating type II DM patients owing to their minimal side effects. However, these AGI drugs still have shortcomings, involving the high cost of production and the need for sugar-like unit(s) in their core structure, and this has motivated researchers to find alternative sources of AGIs [2]. Recently, several plant secondary metabolites of different classes have been investigated as potential candidates for AGI drug development. Interestingly, a great number of phytochemicals obtained from the Orchidaceae family have been found to possess strong *α*-glucosidase inhibitory activity [3,4,5]. Orchidaceae is one of the largest families in the plant kingdom and consists of about 735 genera and more than 20,000 species [6]. Many have been widely used as principal components in traditional Chinese medicine (TCM) and thus have been extensively investigated and found to be a rich source of unusual secondary metabolites [7]. *Gastrochilus*, a small genus of monopodial herbs in Orchidaceae, comprises approximately 62 species, mainly found in Southeast Asia [8]. *Gastrochilus bellinus* (Rchb.f.) Kuntze (“Suea Dam” in Thai and “Wat-Won-Thit-Khwa” in Myanmar) has stems enclosed in basal sheaths of leaves. Its sub-umbellate inflorescence contains small flowers (2–3 cm in diameter), which are distinct morphological features of this genus. The key characteristics of *G. bellinus* are flowers having pale yellow sepals, petals with brownish–purple markings, and a central cushion on white lip epichile with a groove or cavity at the base (Figure 1) [9]. Prior to this study, there were no records on the phytochemical and biological investigation of this plant. As part of our continuing studies on bioactive compounds from orchids [10,11,12], we investigated the chemical constituents of *G. bellinus* and their *α*-glucosidase inhibitory potential. In this study, a dried MeOH extract prepared from *G. bellinus* was suspended in water and partitioned with EtOAc and butanol to give EtOAc, butanol, and aqueous extracts, which were then evaluated for their α-glucosidase inhibitory effect. The EtOAc extract exhibited the strongest activity with 80% inhibition at a concentration of 100 μg/mL and, therefore, was selected for further study. The butanol and aqueous extracts were found to be devoid of activity (less than 10% inhibition).

## 2. Results and Discussion

### 2.1. Structural Characterization

**Figure 2:**
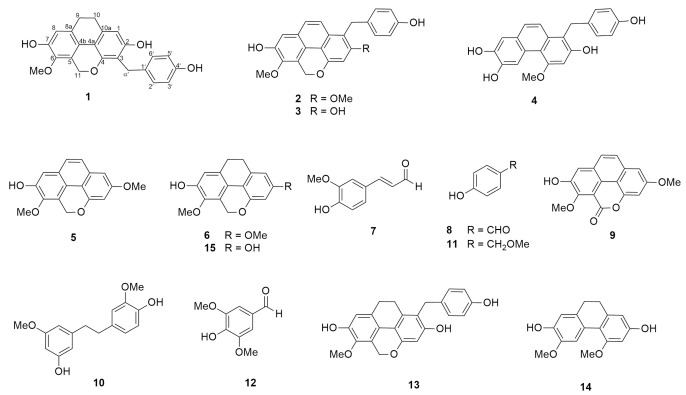
Chemical structures of compounds **1**–**15**.

This study led to the isolation of four hitherto unknown compounds (**1**–**4**) (Supplementary Materials), and eleven known compounds, including agrostophyllin (**5**), agrostophyllidin (**6**), coniferyl aldehyde (**7**), 4-hydroxybenzaldehyde (**8**), agrostophyllone (**9**), gigantol (**10**), 4-(methoxylmethyl)phenol (**11**), syringaldehyde (**12**), 1-(4′-hydroxybenzyl)-imbricartin (**13**), 6-methoxycoelonin (**14**), and imbricatin (**15**) (Figure 2).

**Figure 3:**
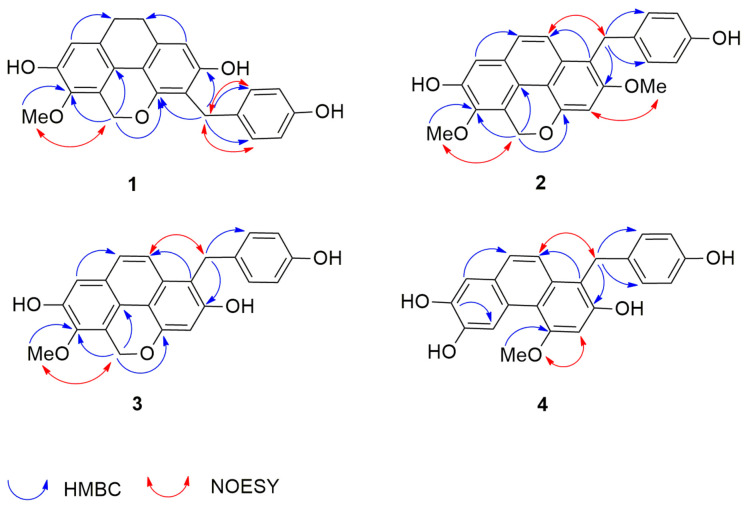
The key HMBC and NOESY correlations of compounds **1**–**4**.

Compound **1** was obtained as a brown amorphous solid. The high-resolution APCI (Atmospheric-pressure chemical ionization) mass spectrum showed a protonated molecular ion [M + H]^+^ at *m/z* 377.1360 (calculated for C_23_H_21_O_5_ 377.1389), suggesting the molecular formula C_23_H_20_O_5_. The IR spectrum displayed bands at 3360, 2921, 1659, and 1633 cm^−1^, indicative of aromatic and hydroxyl functional groups. The UV spectrum of compound **1** showed maximal absorptions at 206 and 284 nm. The ^1^H NMR spectrum (Table 1) suggested a dihydrophenanthropyran structure by the presence of signals for two pairs of methylene protons at δ 2.72 (4H, br s, H_2_-9, H_2_-10), a two-proton singlet at δ 5.17 (2H, s, H_2_-11), and two aromatic proton singlets at δ 6.40 (1H, s, H-1) and 6.69 (1H, s, H-8) [13]. In addition, a resonance for a methoxy group was observed at δ 3.79 (3H, s, 6-OMe). The NMR assignment of H-1 was deduced from its HMBC correlations with C-3 (δ 114.5), C-10 (δ 27.6), and C-4a (δ 111.8). The assignment of H-8 was based on its HMBC correlations with C-6 (δ 141.7), C-9 (δ 27.1), and C-4b (119.6). Apart from the aforementioned signals, the ^1^H NMR spectrum presented resonances for a pair of methylene protons at 3.88 (2H, s, H_2_-α′), and two pairs of two-proton doublets at δ 7.15 (2H, d, *J =* 8.7 Hz, H-2′, H-6′) and 6.66 (2H, d, *J =* 8.7 Hz, H-3′, H-5′), which are characteristic signals of a *p*-hydroxybenzyl group. This was supported by the HMBC correlations of C-α′ (δ 27.6) with H-2′/H-6′ (Figure 3). The ^13^C NMR spectrum (Table 2) showed 21 carbon signals representing 23 carbon atoms. The location of the methoxyl group at C-6 was confirmed by its NOESY interaction with H_2_-11 (Figure 3). In the HMBC spectrum, 3-bond correlations from H_2_-α′ (δ 3.88) to C-4 (δ 150.8), C-2 (δ 154.8) and C-2′/6′ (δ 129.4) connected the 9,10-dihydrophenanthropyran nucleus with the *p*-hydroxybenzyl skeleton at C-3. Based on the above NMR data, compound **1** was determined as a new dihydrophenanthropyran derivative (Figure 2) and given the trivial name gastrobellinol A. The chemical structure of this compound was similar to that of 1-(4′-hydroxybenzyl)-imbricartin (**13**) [14], except that in **1**, the *p*-hydroxybenzyl moiety was located at C-3, instead of C-1, as in **13**. It was observed that C-1 (δ 108.0) of compound **1** resonated at a more downfield position (6.9 ppm) than C-3 (δ 101.1) of **13** when measured in the same NMR solvent (acetone-*d*_6_). The upfield shift of C-3 in **13** was due to the two *ortho*-oxygenations at C-2 and C-4. Moreover, the singlet proton of H-1 of **1** showed HMBC correlation with C-10, whereas no analogous HMBC correlation was observed for H-3 in **13**.

Compound **2** was isolated as a brown amorphous solid. The high-resolution APCI mass spectrum showed a protonated molecular ion [M + H]^+^ at *m/z* 389.1351 (calculated for C_24_H_21_O_5_ 389.1389), suggesting the molecular formula C_24_H_20_O_5_. The UV spectrum of compound **2** (MeOH) showed maximum absorptions at 205, 225, 270, and 380 nm, which were similar to those of a phenanthopyran derivative [15]. The IR spectrum displayed strong absorption bands at 3360, 2921, 1658, and 1633 cm^−1^. The ^1^H NMR spectrum (Table 1) exhibited aromatic protons with *ortho*-coupling at δ 7.55 (1H, d, *J* = 9.3 Hz, H-9) and 7.78 (1H, d, *J* = 9.3 Hz, H-10), and aromatic protons at δ 6.91 (1H, s, H-3) and 7.25 (1H, s, H-8). A pair of oxymethylene protons appeared at δ 5.64 (2H, s, H_2_-11), indicating the presence of a phenanthropyran structure. Moreover, resonances for two methoxy groups were observed at δ 3.94 (3H, s, 2-OMe) and 3.93 (3H, s, 6-OMe). Additional signals for a pair of methylene protons at 4.29 (2H, s, H_2_-α׳) and two pairs of two-proton doublets at δ 7.01 (2H, d, *J* = 8.4 Hz, H-2′, H-6′) and 6.66 (2H, d, *J* = 8.4 Hz, H-3′, H-5′) suggested the presence of a *p*-hydroxybenzyl moiety, which should be located at C-1, as evidenced by the 3-bond coupling between H-2′/H-6′ and C-α′ (δ 28.9). The HMBC connectivities from H_2_-α′ (δ 4.29) to C-2 (δ 156.2), C-10a (δ 129.5), and C-2′/6′ (δ 129.0) supported the position of the *p*-hydroxybenzyl moiety at C-1. In support of this, NOESY correlations (Figure 3) were found from H_2_-α′ (δ 4.29) to H-2′/H6′ and H-10. The methoxy groups were placed at C-2 and C-6 from the NOESY cross-peaks between H-3 (δ 6.91) and 2-OMe protons, and between H_2_-11 (δ 5.64) and 6-OMe protons (δ 3.93). Based on the above NMR data, compound **2** was characterized as a new phenanthropyran derivative possessing a *p*-hydroxybenzyl moiety at C-1 (Figure 2) and named gastrobellinol B.

Compound **3** was collected as a brown amorphous solid. The high-resolution APCI mass spectrum showed a protonated molecular ion [M + H]^+^ at *m/z* 375.1214 (calculated for C_23_H_19_O_5_ 375.1232), suggesting the molecular formula C_23_H_18_O_5_. The UV maximal absorptions at 225, 270, 365, and 380 nm of compound **3** were similar to those of **2**, suggesting the same basic skeleton. The IR spectrum displayed strong absorption bands at 3354, 2925, 1652, and 1614 cm^−1^. The ^1^H and ^13^C NMR (Table 1 and Table 2) and DEPT spectra of compound **3** exhibited signals similar to those of **2**, except that compound **3** had only one methoxy group that showed a cross-peak at δ 3.92 (3H, s, 6-OMe)/δ 60.4 in the HSQC spectrum. The position of the methoxy group at C-6 was deduced from its NOESY cross-peak with H_2_-11 (2H, δ 5.60, s). In the HMBC spectrum (Figure 3), H_2_-11 (δ 5.60) showed a 3-bond correlation with C-6 (δ 143.2), which was also correlated to the 6-OMe protons. The HMBC correlation from H_2_-α′ (2H, δ 4.31, s) to C-2 (δ 153.7), C-10a (δ 129.9), and C-2′/C-6′ (δ 129.1) confirmed the linkage point between the phenanthropyran and the *p-*hydroxybenzyl unit. Based on the aforementioned NMR data, compound **3** was determined to be a de-2-*O*-methyl derivative of **2** (Figure 2) and given the trivial name gastrobellinol C.

Compound **4** was purified as a brown amorphous solid. The high-resolution APCI mass spectrum showed a protonated molecular ion [M + H]^+^ at *m/z* 363.1211 (calculated for C_22_H_19_O_5_ 363.1232), suggesting the molecular formula C_22_H_18_O_5_. The UV spectrum of compound **4** showed maximal absorptions at 230, 265, 355, and 370 nm, suggesting a phenanthrene core structure [16]. The IR spectrum displayed absorption bands at 3360 cm^−1^ for OH, and 2921 and 1658 cm^−1^ for aromatic rings. The ^1^H-NMR spectrum (Table 1) exhibited proton signals similar to those of compound **3**. However, in compound **4**, the signal for the oxymethylene protons of the pyran ring was absent and replaced by a highly deshielded aromatic proton at δ 9.12 (1H, s, H-5). This suggested that compound **4** was a phenanthrene, having a *p*-hydroxybenzyl unit attached to C-1, similar to compound **3**. This was confirmed by the presence of signals for aromatic protons of H-9 (1H, δ 7.48, d, *J* = 9.0 Hz) and H-10 (1H, δ 7.64, d, *J* = 9.0 Hz), and *p-*hydroxybenzyl protons of H-2′/H-6′ (2H, δ 7.03, d, *J* = 8.4 Hz), H-3′/H-5′ (2H, δ 6.66, d, *J* = 8.4 Hz), and H_2_-α′ (δ 4.34). The ^13^C NMR (Table 2) and DEPT spectra showed only one signal for a methylene carbon at (δ 29.4), which was correlated to the methylene protons at 4.34 (2H, s, H_2_-α׳) in the HSQC spectrum. The HMBC spectrum (Figure 3) displayed 3-bond correlations from H-5 (δ 9.12) to C-7 (δ 144.0) and C-8a (δ 126.4), and from H_2_-α′ (δ 4.34) to C-2 (δ 152.4), C-10a (δ 133.3), and C-2′/C-6′ (δ 129.0), confirming the proposed phenanthrene-benzyl skeleton. In the NOESY spectrum (Figure 3), the methoxyl protons at δ 4.06 (3H, s, 4-OMe) displayed a cross-peak with the proton at δ 6.94 (1H, s, H-3). A NOESY correlation (Figure 3) from H_2_-α′ (δ 4.34) with H-10 (1H, δ 7.64, d, *J* = 9.0 Hz) and H-2′/H-6′ was also observed. From all the NMR and MS data, it was concluded that compound **4** was a new phenanthrene with a *p*-hydroxybenzyl substituent (Figure 2), and the trivial name gastrobellinol D was given to the compound.

The other phenolic compounds (Figure 2) were identified by comparison of their spectroscopic data with previous reported data as follows: agrostophyllin (**5**) [15], agrostophyllidin (**6**) [17], coniferyl aldehyde (**7**) [18], 4-hydroxybenzaldehyde (**8**) [19], agrostophyllone (**9**) [17], gigantol (**10**) [20], 4-(methoxylmethyl)phenol (**11**) [21], syringaldehyde (**12**) [22], 1-(4′-hydroxybenzyl)-imbricartin (**13**) [14], 6-methoxycoelonin (**14**) [23], and imbricatin (**15**) [24].

### 2.2. α-Glucosidase Inhibitory Activity

Compounds **1**–**15** were evaluated for their *α*-glucosidase inhibitory activity. Gastrobellinol A (**1**), gastrobellinol B (**2**), gastrobellinol C (**3**), and 1-(4′-hydroxybenzyl)-imbricartin (**13**) showed strong activity with IC_50_ values of 88.72, 97.78, 45.92, and 53.69 µM, respectively, when compared with the drug acarbose (IC_50_ 447.36 µM) (Table 3). Coniferyl aldehyde (**7**), agrostophyllone (**9**), and imbricatin (**15**) also exhibited appreciable activity with IC_50_ values of 380.92, 280.98, and 301.12 µM, respectively. It can be noted herein that for derivatives of phenanthrene or dihydrophenanthrene, the presence of a pyran ring or a *p*-hydroxy benzyl unit is important for *α*-glucosidase inhibitory activity, as reflected by the low IC_50_ values of compounds **1**, **2**, **3**, and **13**. Structures without a *p*-hydroxy benzyl group (compounds **9** and **15**) or a pyran ring (compound **4**) exhibited little or no activity.

Due to its high potency and availability, compound **3** was subjected to a kinetic study to determine the mode of enzyme inhibition. Lineweaver–Burk plots of the inverted values of velocity (1/V) versus the inverted values of substrate concentration (1/[S]) were prepared and analyzed, in comparison with that of acarbose, by varying the concentration (0.25–2.0 mM) of the substrate (*p*NPG) in the presence or absence of compound **3** at two different concentrations (20 and 40 μM).

**Figure 4:**
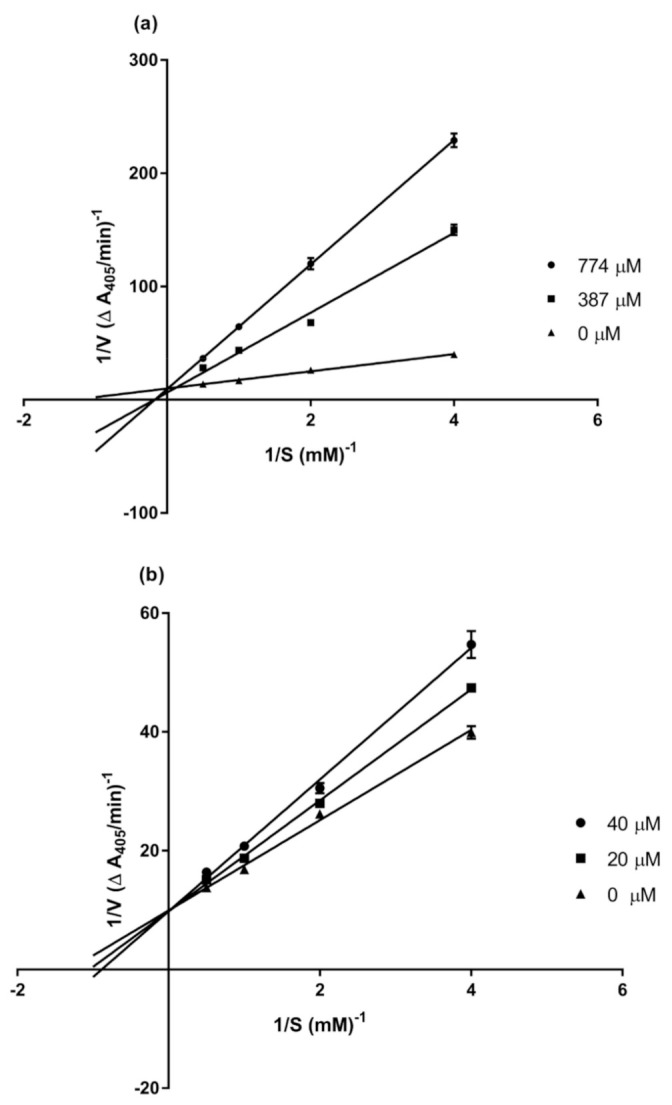
Lineweaver–Burk plots of (**a**) acarbose and (**b**) compound **3**.

The drug acarbose, as expected, showed competitive inhibition, as determined from the Lineweaver–Burk plot (Figure 4a). The secondary plot of acarbose, generated by replotting the slopes of the lines against the inhibitor concentration, gave a K*_i_* value of 143.6 μM (Table 4). The obtained kinetic parameters of compound **3** are listed in Table 4. The maximum velocity (V*_max_*) value was determined as 0.1 A_405/min_, and the Michaelis–Menten constant (K*_m_*) as 0.8, 0.9, and 1.1 µM (Figure 4b). The presence of compound **3** at different concentrations (20 µM and 40 µM) did not change the V*_max_*, but the K*_m_* of the enzyme was increased. These results suggest that **3** is a competitive inhibitor of this enzyme. A secondary plot of **3** gave a K*_i_* value of 87.3 µM. Several phenolic compounds from plants have been earlier reported as competitive inhibitors of *α*-glucosidase, for example, dihydrobenzoxanthones from *Artocarpus elasticus* [25] and flavonoids from *Agrimonia pilosa* [26].

## 3. Materials and Methods

### 3.1. General Experimental Procedures

UV spectroscopic data were determined using an Agilent Cary 60 Spectrophotometer (Penang, PG, Malaysia), and the IR data were obtained via a Perkin–Elmer FT-IR 1760x spectrophotometer (Boston, MA, USA). High-resolution Atmospheric Pressure Chemical Ionization mass spectra (HR-APCI-MS) were recorded with a Bruker micro TOF-QII mass spectrometer (Billerica, MA, USA). ^1^H and ^13^C NMR spectra were recorded with a Bruker Avance DPX-300 (Billerica, MA, USA).

### 3.2. Plant Material

The whole plants of *Gastrochilus bellinus* were purchased from Chatuchak market, Bangkok, in March 2018. Plant identification was done by one of the authors (B. Sritularak) and compared with the database of the Botanical Garden Organization. A voucher specimen (BS-GBel-032561) has been deposited at the herbarium of the Department of Pharmacognosy and Pharmaceutical Botany, Faculty of Pharmaceutical Sciences, Chulalongkorn University.

### 3.3. Extraction and Isolation

The air-dried samples of *Gastrochilus bellinus* (3.6 kg) were chopped and extracted with methanol (MeOH) to obtain a MeOH extract after removal of the solvent. The MeOH extract (750 g) was suspended in water and partitioned with ethyl acetate (EtOAc) and *n*-butanol (BuOH) to get an EtOAc, a BuOH, and an aqueous extract after drying. The EtOAc extract (60 g) was further fractionated by vacuum–liquid chromatography (VLC) on silica gel (CH_2_Cl_2_-EtOAc, gradient up to 4:6, followed by CH_2_Cl_2_–acetone, isocratic, 1:1) to give three fractions (A–C). Fraction A (32.2 g) was separated by column chromatography (CC, silica gel CH_2_Cl_2_-EtOAc, isocratic, 9.8:0.2) to give eight fractions (AI–AVIII). Fraction AI (560 mg) was separated again by CC (silica gel, CH_2_Cl_2_-EtOAc, isocratic, 9.8:0.2) to obtain five fractions (AI_1_–AI_5_). Fraction AI_3_ was subjected to column chromatography (CC, silica gel, hexane–EtOAc, gradient) followed by Sephadex LH-20 (acetone) to yield agrostophyllin (**5**) (27.2 mg) and agrostophyllidin (**6**) (4.1 mg). Fraction AI_4_ was purified on Sephadex LH-20 (acetone) to yield coniferyl aldehyde (**7**) (5.3 mg). Fraction AI_5_ was separated on Sephadex LH-20 (acetone) to give AI_5a_ to AI_5h_. From fraction AI_5c_, 4-hydroxybenzaldehyde (**8**) (50.2 mg), Agrostophyllone (**9**) (7 mg), and compound **1** (2.2 mg) were obtained by purifying on silica gel column (hexane–EtOAc, isocratic, 7:3). Compound **2** (5.2 mg) was isolated from fraction AI_5d_ by purifying on CC (hexane–EtOAc, isocratic, 7:3). Gigantol (**10**) (20.4 mg), 4-(methoxylmethyl)phenol (**11**) (73.3 mg), and syringaldehyde (**12**) (5.6 mg) were collected from fraction AI_5f_ by using reverse phase C-18 CC (MeOH–water, gradient). Fraction AI_5h_ was purified on CC (silica gel, CH_2_Cl_2_–EtOAc, isocratic, 9.8:0.2) to afford 1-(4′-hydroxybenzyl)-imbricartin (**13**) (9.9 mg). Fraction AIII (105.7 mg) was separated on Sephadex LH-20 (acetone) to give four fractions (AIII_1_ to AIII_4_). AIII_2_ was purified on Sephadex LH-20 (MeOH) to get 6-methoxycoelonin (**14**) (6.9 mg). Compound **3** (10.5 mg) and compound **4** (3.6 mg) were obtained from fraction AIII_3_ by purifying on CC (silica gel, hexane–acetone, gradient). Imbricatin (**15**) (57.3 mg) was collected from fraction AV by purifying on Sephadex LH-20 (CH_2_Cl_2_–MeOH, 1:1).

Gastrobellinol A (**1**): Brown amorphous solid; UV (MeOH): λmax (log ε) 206 (4.76), 284 (4.34) nm; IR: ν_max_ 3360, 2921, 2851, 1659 and 1633 cm^−1^; HR-APCI-MS: [M + H]^+^ *m/z* 377.1360 (calculated for C_23_H_21_O_5_ 377.1389); ^1^H and ^13^C NMR data, see Table 1 and Table 2.

Gastrobellinol B (**2**): Brown amorphous solid; UV (MeOH): λmax (log ε) 205 (4.37), 225 (4.40), 270 (4.42), 380(3.49) nm; IR: ν_max_ 3360, 2921, 2850, 1658 and 1633 cm^−1^; HR-APCI-MS: [M + H]^+^ at *m/z* 389.1351 (calculated for C_24_H_21_O_5_ 389.1389); ^1^H and ^13^C NMR data, see Table 1 and Table 2.

Gastrobellinol C (**3**): Brown amorphous solid; UV (MeOH): λmax (log ε) 225 (4.61), 270 (4.52), 365 (3.62), 380(3.64) nm; IR: ν_max_ 3354, 2925, 2853, 1652 and 1614 cm^−1^; HR-APCI-MS: [M + H]^+^ *m/z* 375.1214 (calculated for C_23_H_19_O_5_ 375.1232); ^1^H and ^13^C NMR data, see Table 1 and Table 2.

Gastrobellinol D (**4**): Brown amorphous solid; UV (MeOH): λmax (log ε) 230 (3.79), 265 (3.82), 255 (3.96), and 370 (3.12) nm; IR: ν_max_ 3360, 2921, 2851, 1658, 1632 cm^−1^; HR-APCI-MS: [M + H]^+^ *m/z* 363.1211 (calculated for C_22_H_19_O_5_ 363.1232); ^1^H and ^13^C NMR data, see Table 1 and Table 2.

### 3.4. α-Glucosidase Inhibitory Assay

The *α*-glucosidase enzyme inhibition assay was carried out according to the method in our previous report [27]. The inhibitory activity was determined by measuring the *p*-nitrophenol, a yellow color substance that can be monitored at 405 nm, released from *p*-nitrophenyl-α-D-glucopyranoside (*p*NPG) by the *α*-glucosidase enzyme. The test samples were initially dissolved in 50% DMSO, and then, 10 µL of the sample solution and 40 µL of 0.1 unit/mL α-glucosidase were incubated at 37 °C for 10 min. After that, to start the reaction, 50 µL of 2 mM *p*NPG was added to the mixture and incubated at 37 °C for 20 min. One hundred microliters of 1 M Na_2_CO_3_ was added to stop the reaction, and then, the absorbance was measured at 405 nm. Acarbose was used as a positive control, and 5% DMSO was used as a negative control. Each experiment was performed in triplicate. Data were displayed as mean ± SD.

The enzyme kinetics parameters (K*_m_* and V*_max_*) were determined by analyzing the double reciprocal Lineweaver–Burk plot (1/V vs. 1/[S]). Each experiment was carried out by varying the concentration of *p*NPG (2.0, 1.0, 0.5, and 0.25 mM) in the absence and presence of different concentrations of the test sample. The reaction was monitored at 405 nm by a microplate reader every 5 min for a total time of 25 min. Each experiment was performed in triplicate. Acarbose and 5% DMSO served as the positive and negative controls, respectively. A secondary plot for acarbose was generated by plotting the slopes of the double-reciprocal lines versus inhibitor concentration [28]. For compound **3**, secondary plots were plots of the inverted values of K*_m_* (1/K*_m_*) as a function of inhibitor concentration. The inhibition constant (K*_i_*) was then calculated from the intersection point.

## 4. Conclusions

This study is the first report on the secondary metabolites of *Gastrochilus bellinus*. In summary, the phytochemical investigation of *Gastrochilus bellinus* led to the isolation of four new compounds gastrobellinols A-D (**1–4**), along with eleven known compounds. When isolates were determined for α-glucosidase inhibitory activity, compounds **1**, **2**, **3**, **7**, **8**, **13**, and **15** showed higher α-glucosidase inhibitory activity than acarbose. The potent α-glucosidase inhibitor, compound **3**, revealed its competitive inhibition on the α-glucosidase enzyme.

## Figures and Tables

**Table 1:** ^1^H (300 MHz) NMR data of compounds **1**–**4** in acetone-*d*_6_.

Position	1	2	3	4
1	6.40, s	-	-	-
2	-	-	-	-
3	-	6.91, s	6.79, s	6.94, s
4	-	-	-	-
4a	-	-	-	-
4b	-	-	-	-
5	-	-	-	9.12, s
6	-	-	-	-
7	-	-	-	-
8	6.69, s	7.25, s	7.23, s	7.21, s
8a	-	-	-	-
9	2.72, br s	7.55, d (9.3)	7.53, d (9.3)	7.48, d (9.0)
10	2.72, br s	7.78, d (9.3)	7.75, d (9.3)	7.64, d (9.0)
10a	-	-	-	-
11	5.17, s	5.64, s	5.60, s	
α′	3.88, s	4.29, s	4.31, s	4.34, s
1′	-	-	-	-
2′	7.15, d (8.7)	7.01, d (8.4)	7.07, d (8.4)	7.03, d (8.4)
3′	6.66, d (8.7)	6.66, d (8.4)	6.67, d (8.4)	6.66, d (8.4)
4′	-	-	-	-
5′	6.66, d (8.7)	6.66, d (8.4)	6.67, d (8.4)	6.66, d (8.4)
6′	7.15, d (8.7)	7.01, d (8.4)	7.07, d (8.4)	7.03, d (8.4)
2-OMe	-	3.94, s	-	-
4-OMe	-	-	-	4.06, s
6-OMe	3.79, s	3.93, s	3.92, s	-

**Table 2:** ^13^C (75 MHz) NMR data of compounds **1**–**4** in acetone-*d*_6_.

Position	1	2	3	4
1	108.0	116.1	114.0	113.2
2	154.8	156.2	153.7	152.4
3	114.5	98.2	101.8	98.7
4	150.8	151.5	151.0	157.7
4a	111.8	112.1	111.9	114.9
4b	119.6	118.1	118.4	125.3
5	121.3	120.1	119.9	112.8
6	141.7	143.3	143.2	145.2
7	148.4	149.6	149.4	144.0
8	114.9	110.9	110.7	111.4
8a	128.6	125.2	125.0	126.4
9	27.1	125.8	125.5	127.1
10	27.6	122.6	122.6	120.5
10a	132.4	129.5	129.9	133.3
11	63.3	63.8	63.7	-
α′	27.6	28.9	29.0	29.4
1′	132.6	132.4	132.6	132.6
2′	129.4	129.0	129.1	129.0
3′	114.6	114.9	114.9	114.8
4′	155.2	155.2	155.2	155.2
5′	114.6	114.9	114.9	114.8
6′	129.4	129.0	129.1	129.0
2-OMe	-	55.8	-	-
4-OMe	-	-	-	54.9
6-OMe	60.4	60.4	60.4	-

**Table 3:** *α*-Glucosidase inhibitory activity of compounds **1**–**15**.

Compound	IC_50_ (μM)
Gastrobellinol A (**1**)	88.72 ± 4.1
Gastrobellinol B (**2**)	97.78 ± 3.1
Gastrobellinol C (**3**)	45.92 ± 2.8
Gastrobellinol D (**4**)	NA
Agrostophyllin (**5**)	NA
Agrostophyllidin (**6**)	NA
Coniferyl aldehyde (**7**)	380.92 ± 9.3
4-Hydroxybenzaldehyde (**8**)	NA
Agrostophyllone (**9**)	280.98 ± 15.9
Gigantol (**10**)	NA
4-(Methoxylmethyl)phenol (**11**)	NA
Syringaldehyde (**12**)	NA
1-(4′-Hydroxybenzyl)-imbricartin (**13**)	53.69 ± 12.5
6-Methoxycoelonin (**14**)	NA
Imbricatin (**15**)	301.12 ± 6.6
Acarbose	447.36 ± 28.3

NA means no inhibitory activity.

**Table 4:** Kinetic parameters of *α*-glucosidase inhibition in the presence of compound **3**.

Inhibitor	Dose (µM)	V*_max_* (∆ A_405/min_)	K*_m_* (mM)	K*_i_* (µM)
None	-	0.1	0.8	
Compound **3**	20	0.1	0.9	87.3
	40	0.1	1.1	
Acarbose	387	0.1	4.8	143.6
	744	0.1	6.5	

## Data Availability

All data presented in this study are available in the article and in the Supplementary Material.

## Supplementary Materials

Figure S1: APCI-MS spectrum of compound **1**, Figure S2: ^1^H NMR (acetone-*d*_6_, 300 MHz) spectrum of compound **1**, Figure S3: ^13^C NMR and DEPT (acetone-*d*_6_, 75 MHz) spectrum of compound **1**, Figure S4: HSQC spectrum of compound **1**, Figure S5: HMBC spectrum of compound **1**, Figure S6: NOESY spectrum of compound **1**, Figure S7: APCI-MS spectrum of compound **2**, Figure S8: ^1^H NMR (acetone-*d*_6_, 300 MHz) spectrum of compound **2**, Figure S9: ^13^C NMR and DEPT (acetone-*d*_6_, 75 MHz) spectrum of compound **2**, Figure S10: HSQC spectrum of compound **2**, Figure S11: HMBC spectrum of compound **2**, Figure S12: NOESY spectrum of compound **2**, Figure S13: APCI-MS spectrum of compound **3**, Figure S14: ^1^H NMR (acetone-*d*_6_, 300 MHz) spectrum of compound **3**, Figure S15: ^13^C NMR and DEPT (acetone-*d*_6_, 75 MHz) spectrum of compound **3**, Figure S16: HSQC spectrum of compound **3**, Figure S17: HMBC spectrum of compound **3**, Figure S18: NOESY spectrum of compound **3**, Figure S19: APCI-MS spectrum of compound **4**, Figure S20: ^1^H NMR (acetone-*d*_6_, 300 MHz) spectrum of compound **4**, Figure S21: ^13^C NMR and DEPT (acetone-*d*_6_, 75 MHz) spectrum of compound **4**, Figure S22: HSQC spectrum of compound **4**, Figure S23: HMBC spectrum of compound **4**, Figure S24: NOESY spectrum of compound **4**.
