# Supplementary material for: Four Novel Phenanthrene Derivatives with α-Glucosidase Inhibitory Activity from Gastrochilus bellinus
Source: Molecules. 2021 Jan 14;26(2):418. doi: 10.3390/molecules26020418 (PMC7830893; doi:10.3390/molecules26020418)
Supplement: Supplementary file 1 [file molecules-26-00418-s001.pdf]

Supporting Information

Four Novel Phenanthrene Derivatives with *α*-Glucosidase Inhibitory Activity from *Gastrochilus bellinus*

Htoo Tint San [1,2], Nutputsorn Chatsumpun [3], Thaweesak Juengwatanatrakul [4], Natapol Pornputtapong [5], Kittisak Likhitwitayawuid [1] and Boonchoo Sritularak [1,6,*]

1 Department of Pharmacognosy and Pharmaceutical Botany, Faculty of Pharmaceutical Sciences, Chulalongkorn University, Bangkok 10330, Thailand; htootintsan@mohs.edu.mm (H.T.S.); Kittisak.L@chula.ac.th (K.L.)
2 Permanent address: Department of Pharmacognosy, University of Pharmacy, Yangon 11031, Myanmar
3 Department of Pharmacognosy, Faculty of Pharmacy, Mahidol University, Bangkok, 10400, Thailand; nutputsorn.cha@mahidol.ac.th (N.C.)
4 Faculty of Pharmaceutical Sciences, Ubon Ratchathani University, Ubon Ratchathani, 34190, Thailand; thaweesak.j@ubu.ac.th (T.J.)
5 Department of Biochemistry and Microbiology, Faculty of Pharmaceutical Sciences, Chulalongkorn University, Bangkok 10330, Thailand; Natapol.P@chula.ac.th (N.P.)
6 Natural Products for Ageing and Chronic Diseases Research Unit, Faculty of Pharmaceutical Sciences, Chulalongkorn University, Bangkok, 10330, Thailand

TABLE OF CONTENTS

Figure 1S. APCI-MS spectrum of compound **1**. 3

Figure 2S. ^{1}H NMR (acetone-d_6, 300 MHz) spectrum of compound **1**. 4

Figure 3S. ^{13}C NMR and DEPT (acetone-d_6, 75 MHz) spectrum of compound **1**. 5

Figure 4S. HSQC (acetone-d_6, 300/75 MHz) spectrum of compound **1** 6

Figure 5S. HMBC (acetone-d_6, 300/75 MHz) spectrum of compound **1**. 7

Figure 6S. NOESY (acetone-d_6, 300 MHz) spectrum of compound **1**. 8

Figure 7S. APCI-MS spectrum of compound **2**. 9

Figure 8S. ^{1}H NMR (acetone-d_6, 300 MHz) spectrum of compound **2** 10

Figure 9S. ^{13}C NMR and DEPT (acetone-d_6, 75 MHz) spectrum of compound **2**. 11

Figure 10S. HSQC (acetone-d_6, 300/75 MHz) spectrum of compound **2**. 12

Figure 11S. HMBC (acetone-d_6, 300/75 MHz) spectrum of compound **2**. 13

Figure 12S. NOESY (acetone-d_6, 300 MHz) spectrum of compound **2**. 15

Figure 13S. APCI-MS spectrum of compound **3**. 15

Figure 14S. ^{1}H NMR (acetone-d_6, 300 MHz) spectrum of compound **3** 16

Figure 15S. ^{13}C NMR and DEPT (acetone-d_6, 75 MHz) spectrum of compound **3**. 17

Figure 16S. HSQC (acetone-d_6, 300/75 MHz) spectrum of compound **3** 18

Figure 17S. HMBC (acetone-d_6, 300/75 MHz) spectrum of compound **3**. 19

Figure 18S. NOESY (acetone-d_6, 300 MHz) spectrum of compound **3**. 20

Figure 19S. APCI-MS spectrum of compound **4**. 21

Figure 20S. ^{1}H NMR (acetone-d_6, 300 MHz) spectrum of compound **4** 22

Figure 21S. ^{13}C NMR and DEPT (acetone-d_6, 75 MHz) spectrum of compound **4**. 23

Figure 22S. HSQC (acetone-d_6, 300/75 MHz) spectrum of compound **4**. 24

Figure 23S. HMBC (acetone-d_6, 300/75 MHz) spectrum of compound **4**. 25

Figure 24S. NOESY (acetone-d_6, 300 MHz) spectrum of compound **4**. 26

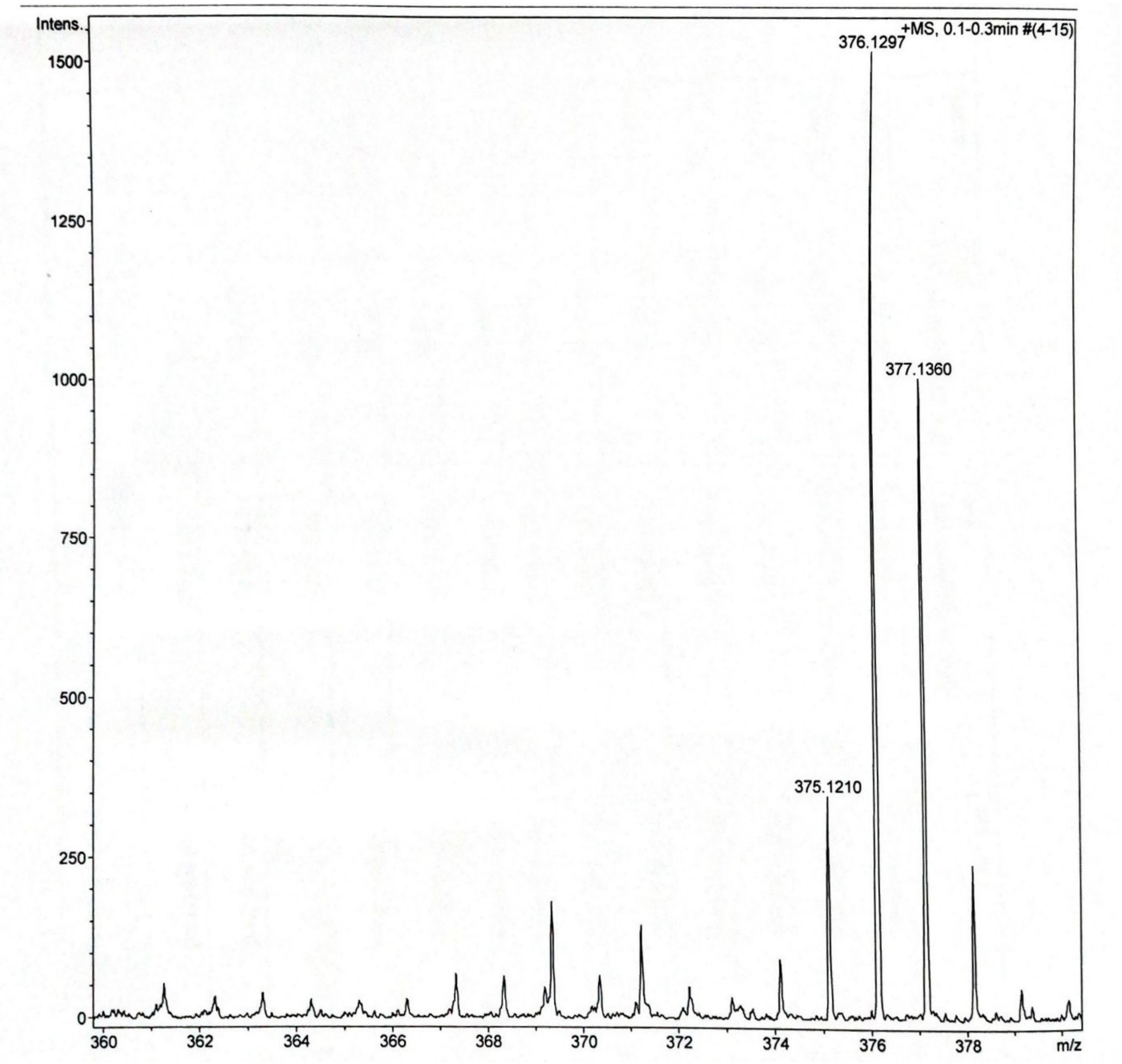

Figure 1S. APCI-MS spectrum of compound 1.

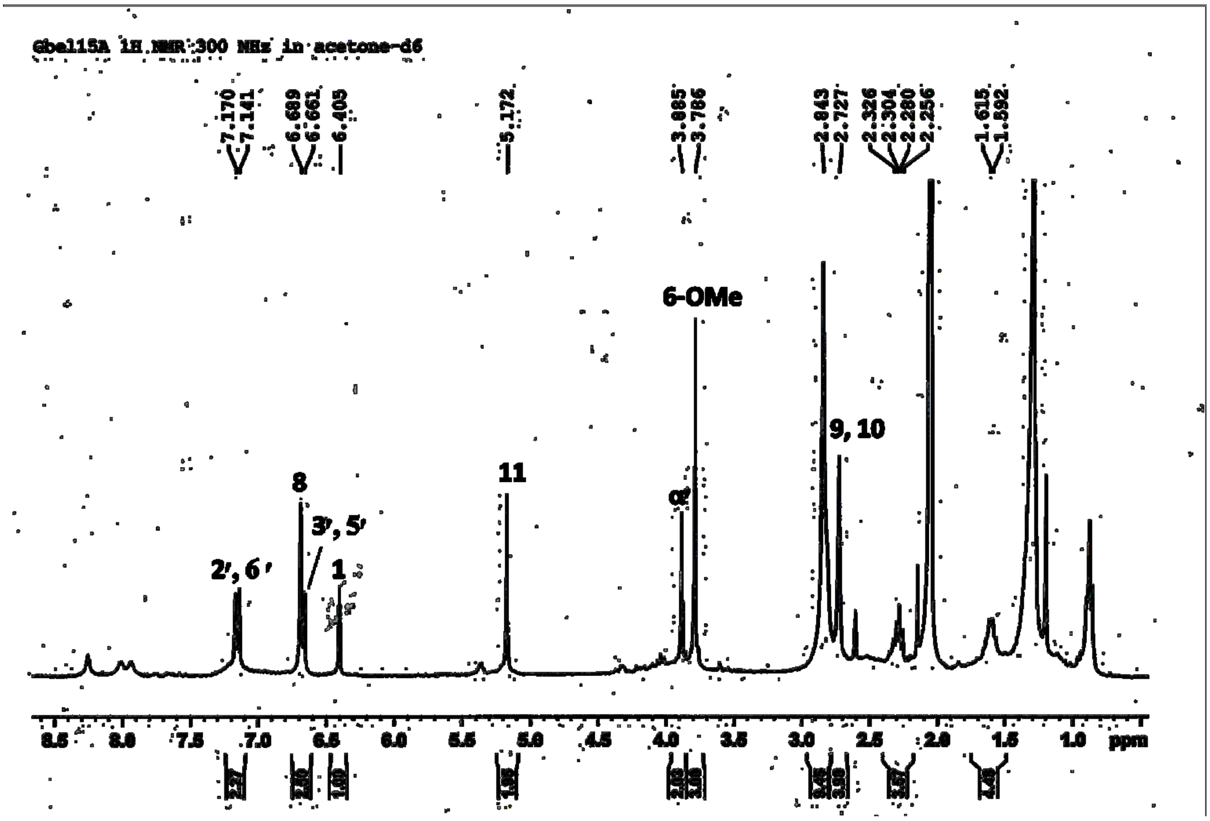

Figure 2S. ^{1}H NMR (acetone-d_6, 300 MHz) spectrum of compound 1.

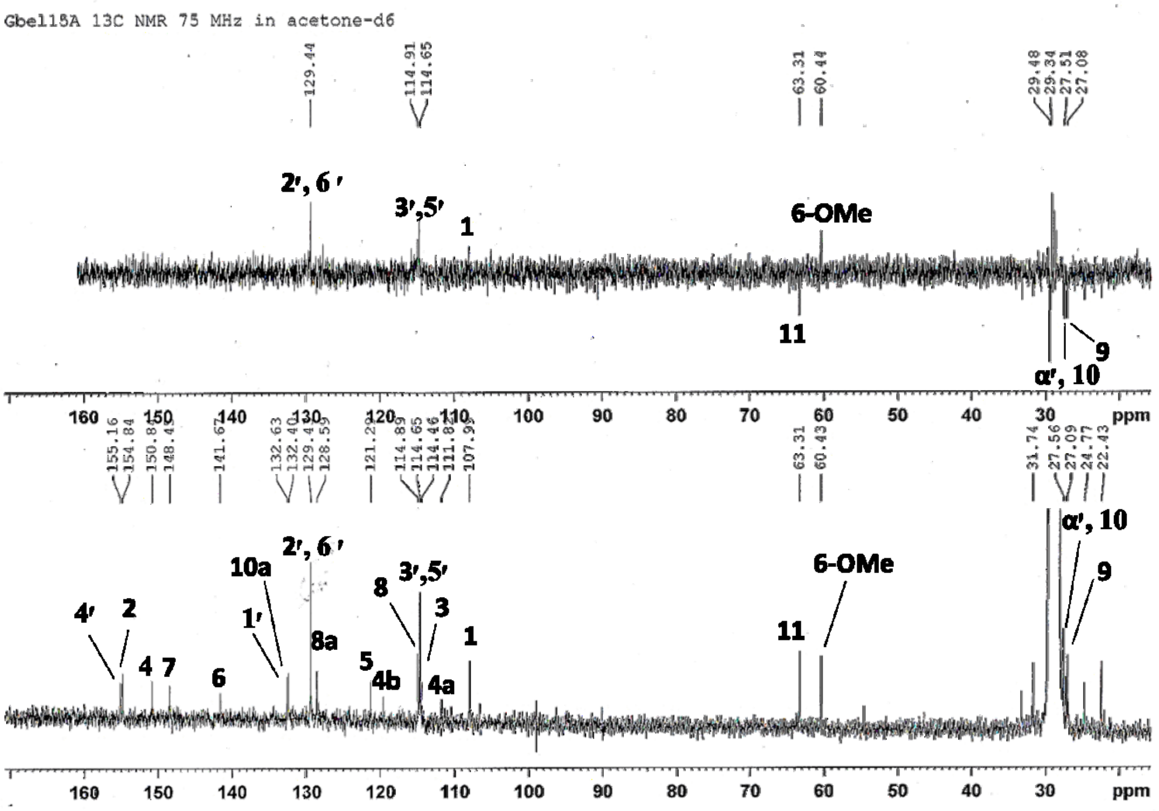

Figure 3S. ^{13}C NMR and DEPT (acetone-d_6, 75 MHz) spectrum of compound 1.

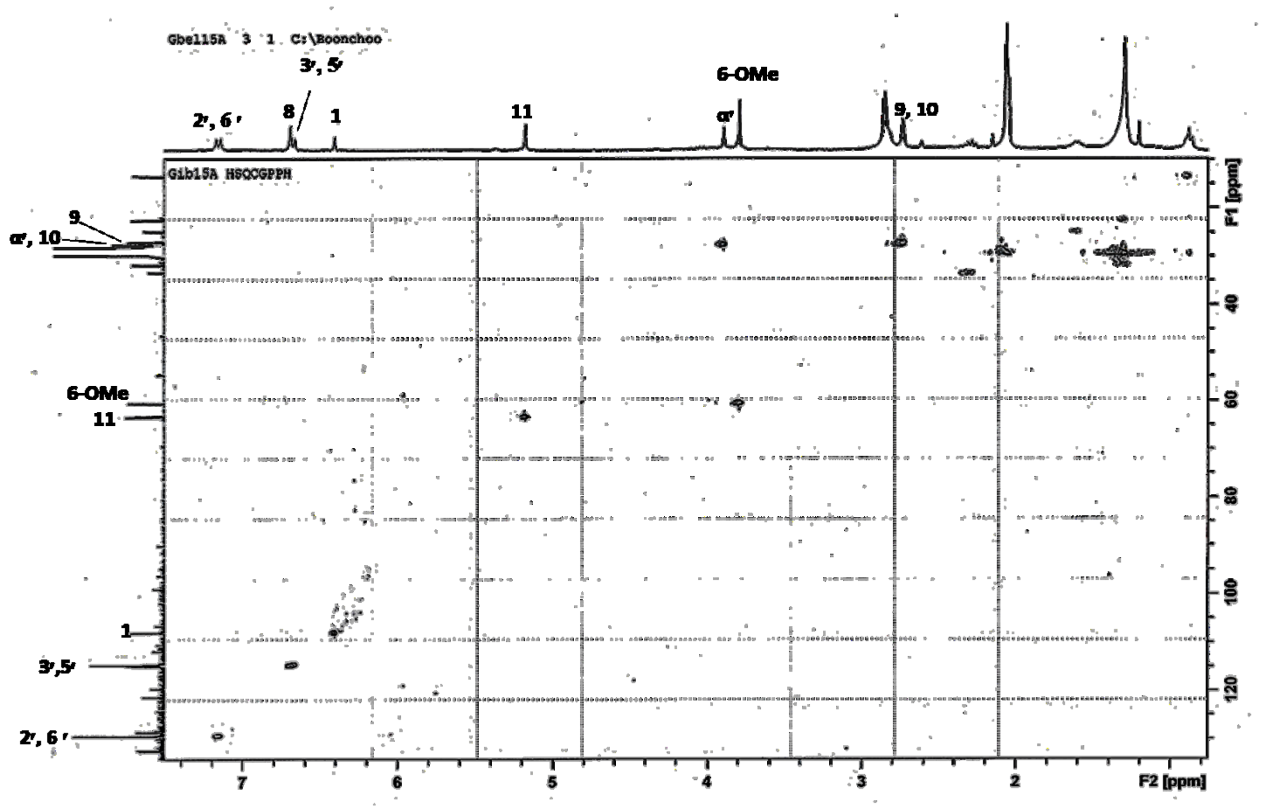

Figure 4S. HSQC (acetone-d_6, 300/75 MHz) spectrum of compound 1.

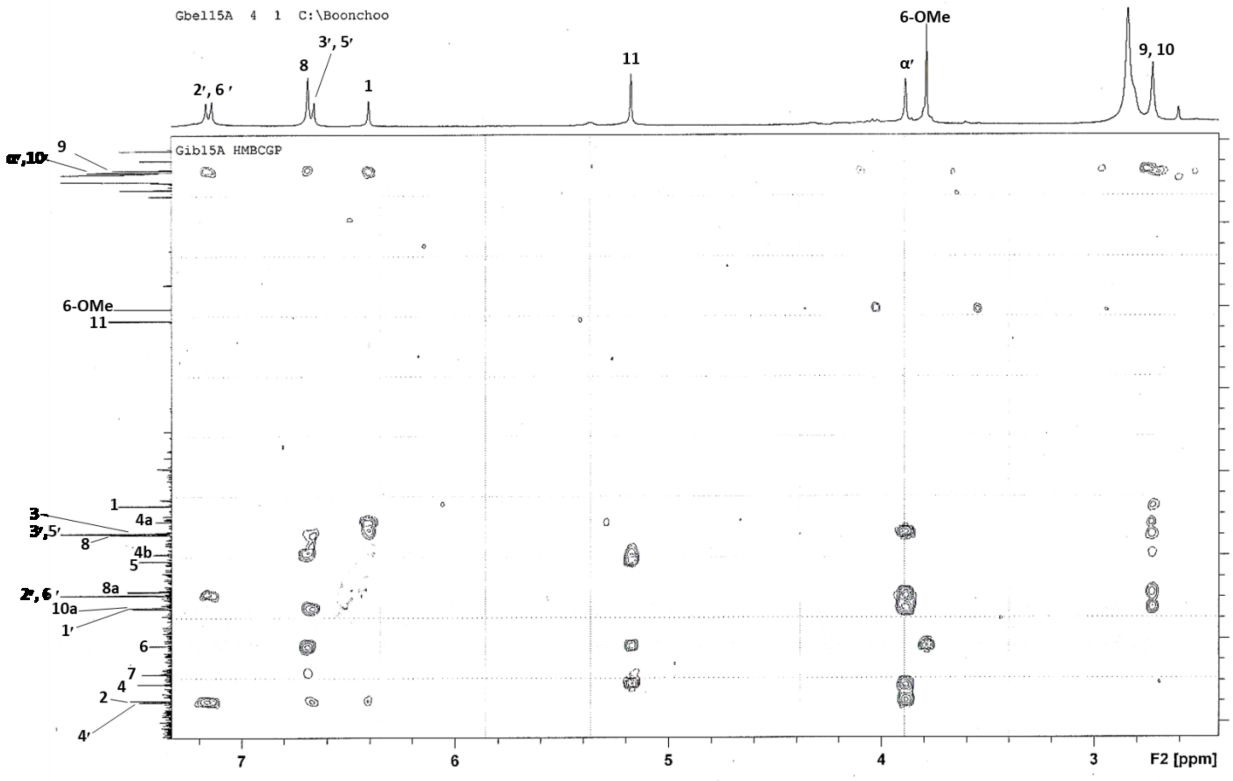

Figure 5S. HMBC (acetone-d_6, 300/75 MHz) spectrum of compound 1.

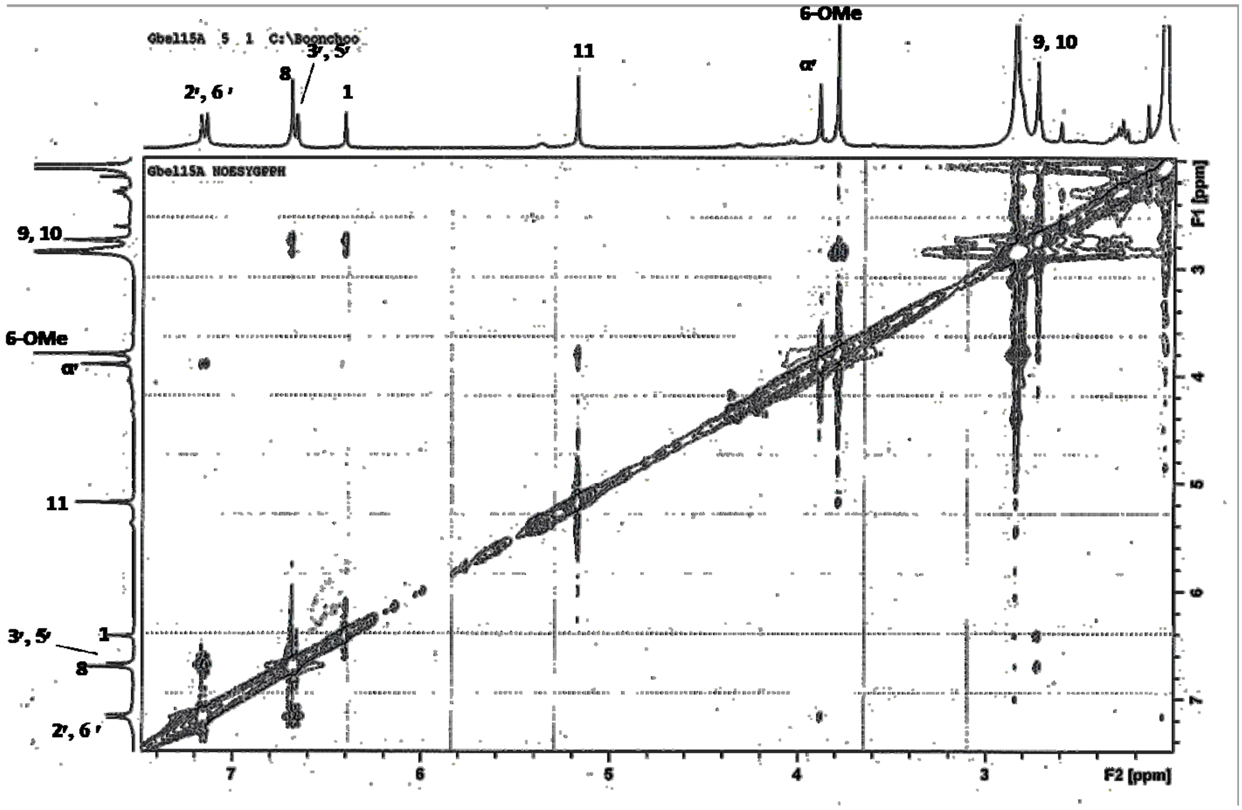

Figure 6S. NOESY (acetone-d_6, 300 MHz) spectrum of compound 1.

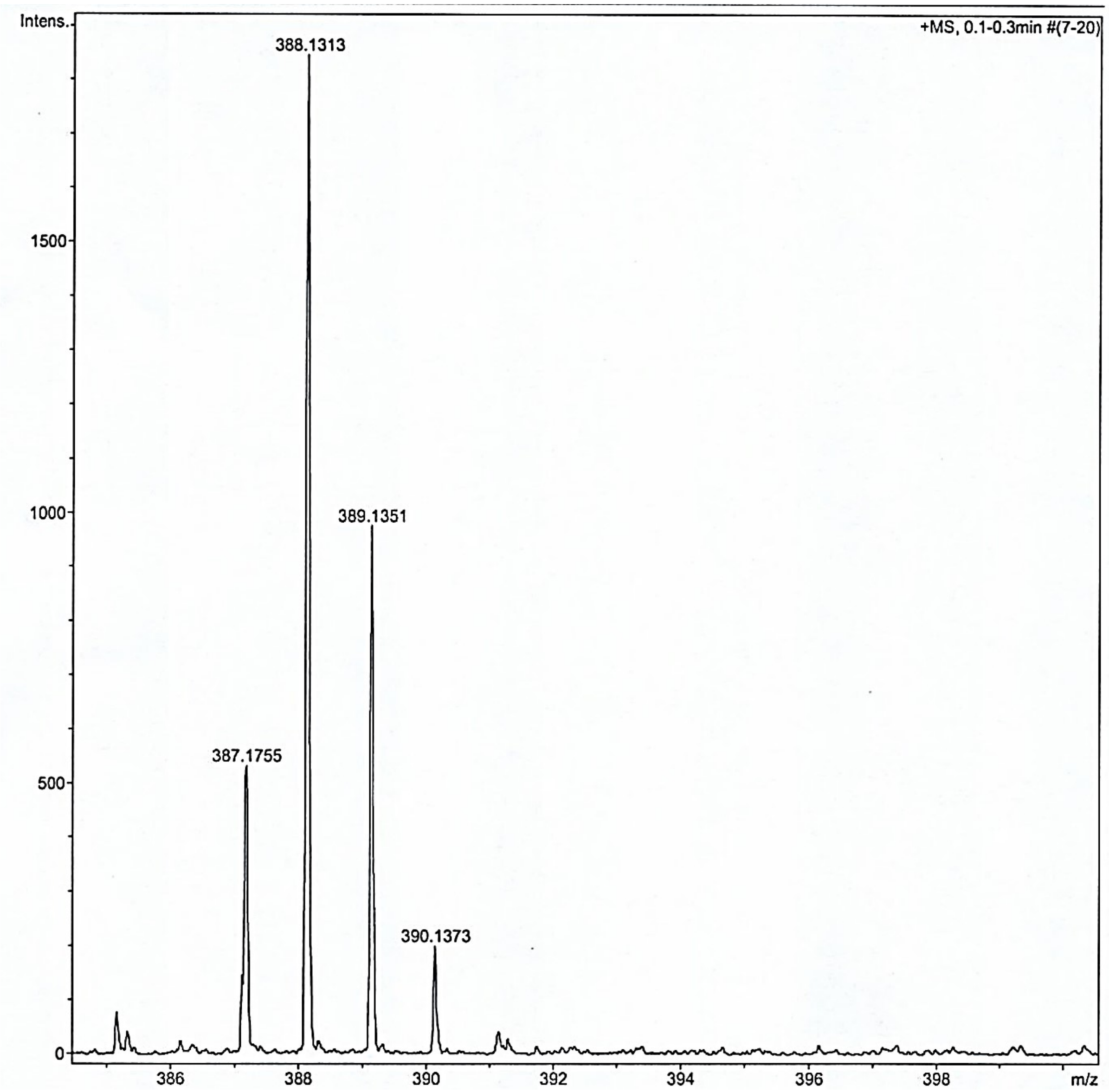

Figure 7S. APCI-MS spectrum of compound 2.

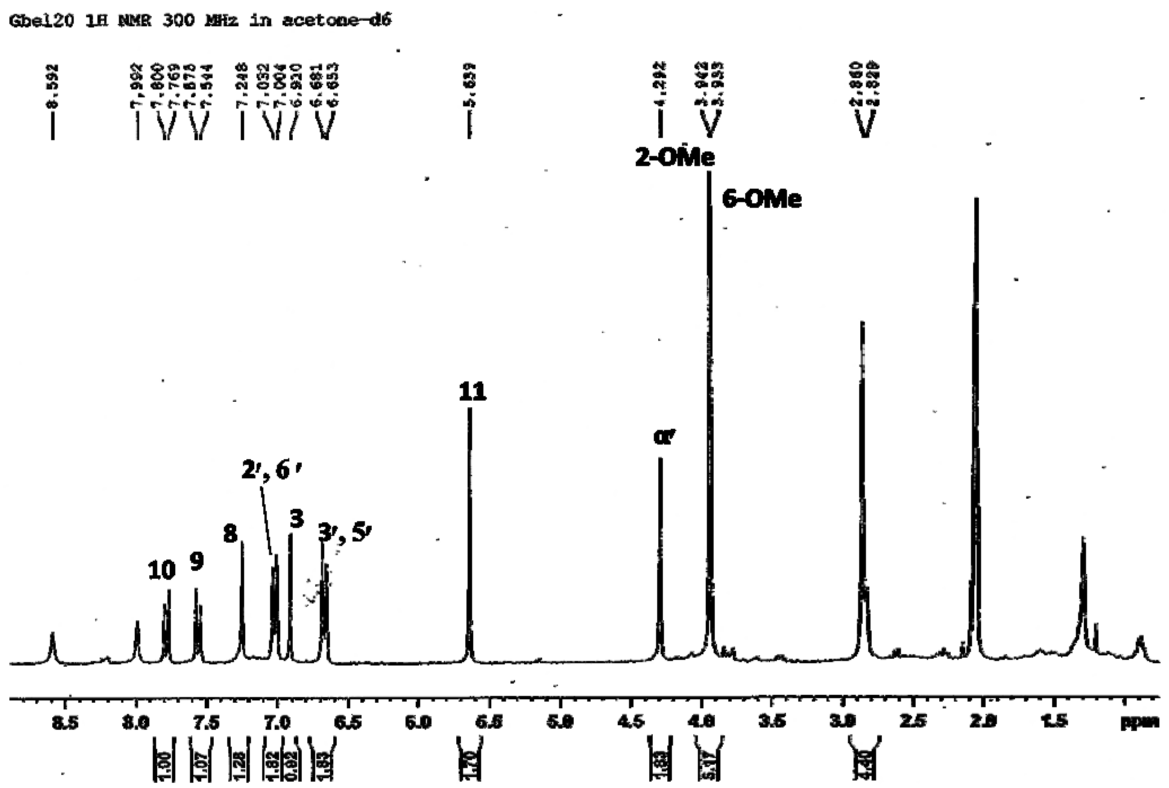

Figure 8S. ^{1}H NMR (acetone-d_6, 300 MHz) spectrum of compound 2.

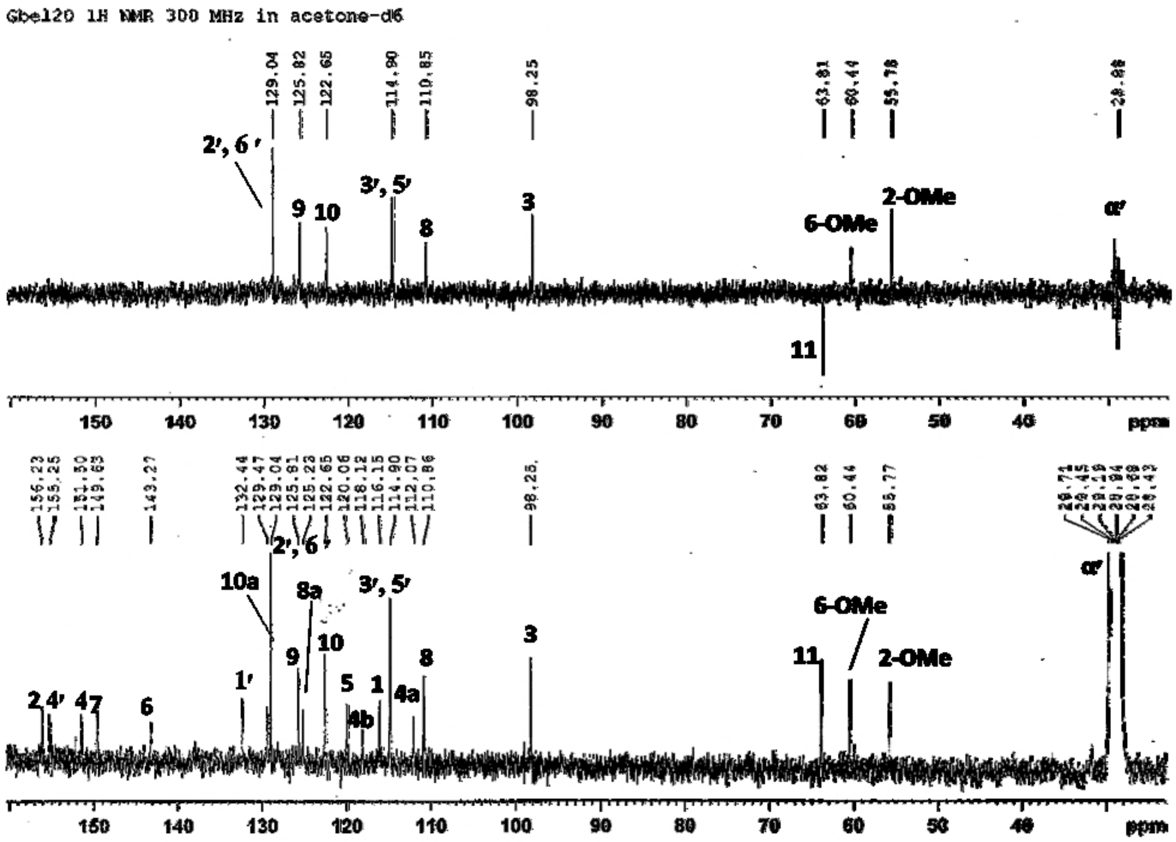

Figure 9S. ^{13}**C NMR and DEPT (acetone-*d_6*, 75 MHz) spectrum of compound 2.**

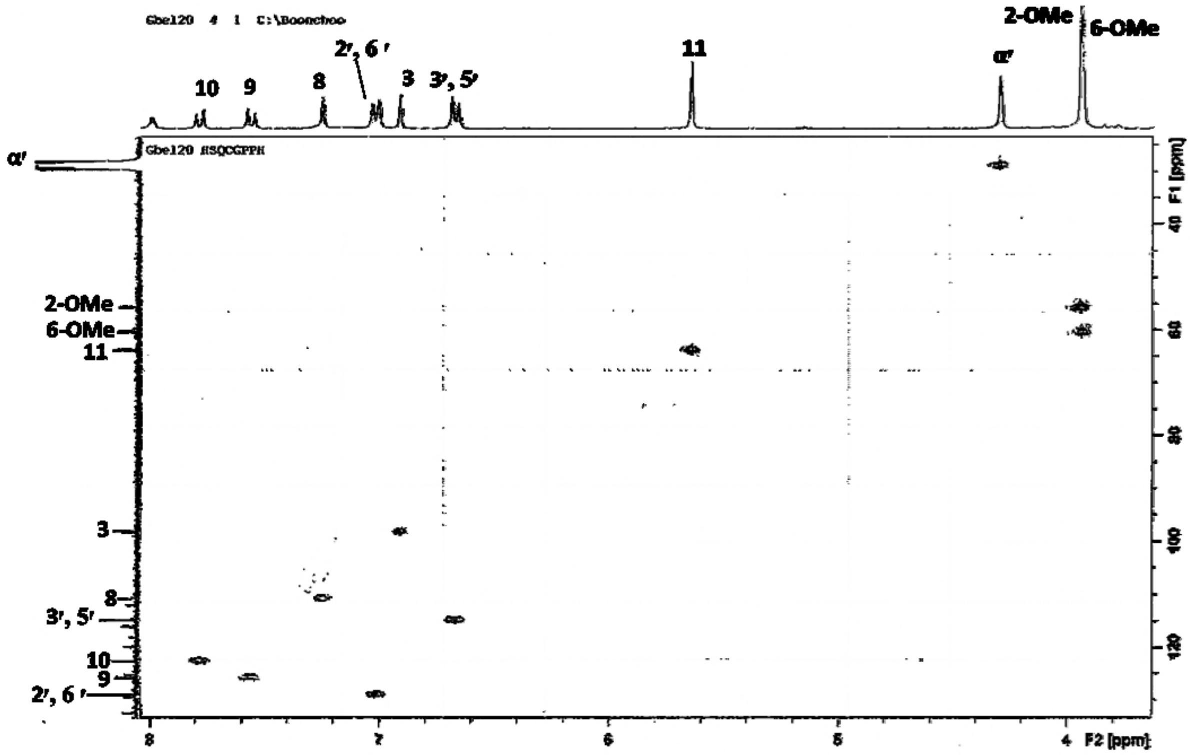

Figure 10S. HSQC (acetone-d_6, 300/75 MHz) spectrum of compound 2.

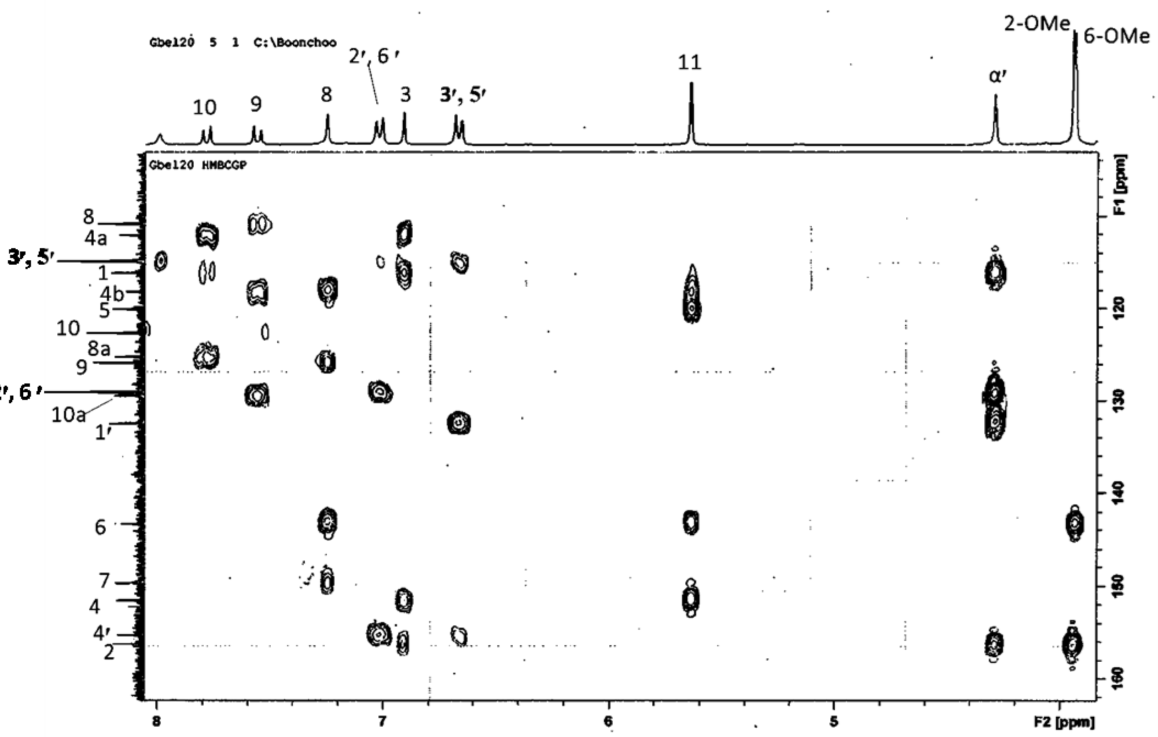

Figure 11S. HMBC (acetone-d_6, 300/75 MHz) spectrum of compound 2.

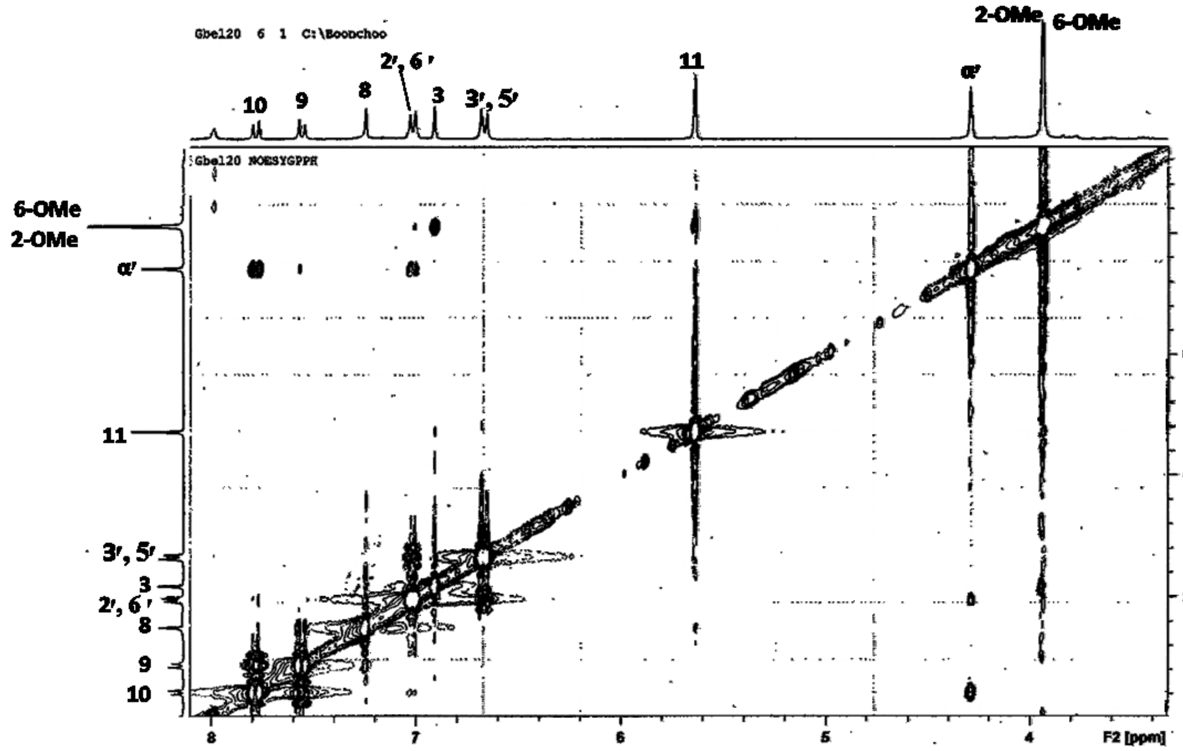
Gbel20 6 1 C:\Boonchoo
Gbel20 NOESYGPPH
10
9
8
2′, 6′
3
3′, 5′
11
α′
2-OMe
6-OMe
6-OMe
2-OMe
α′
11
3′, 5′
3
2′, 6′
8
9
10
F2 [ppm]
F1 [ppm]

Figure 12S. NOESY (acetone-d_6, 300 MHz) spectrum of compound 2.

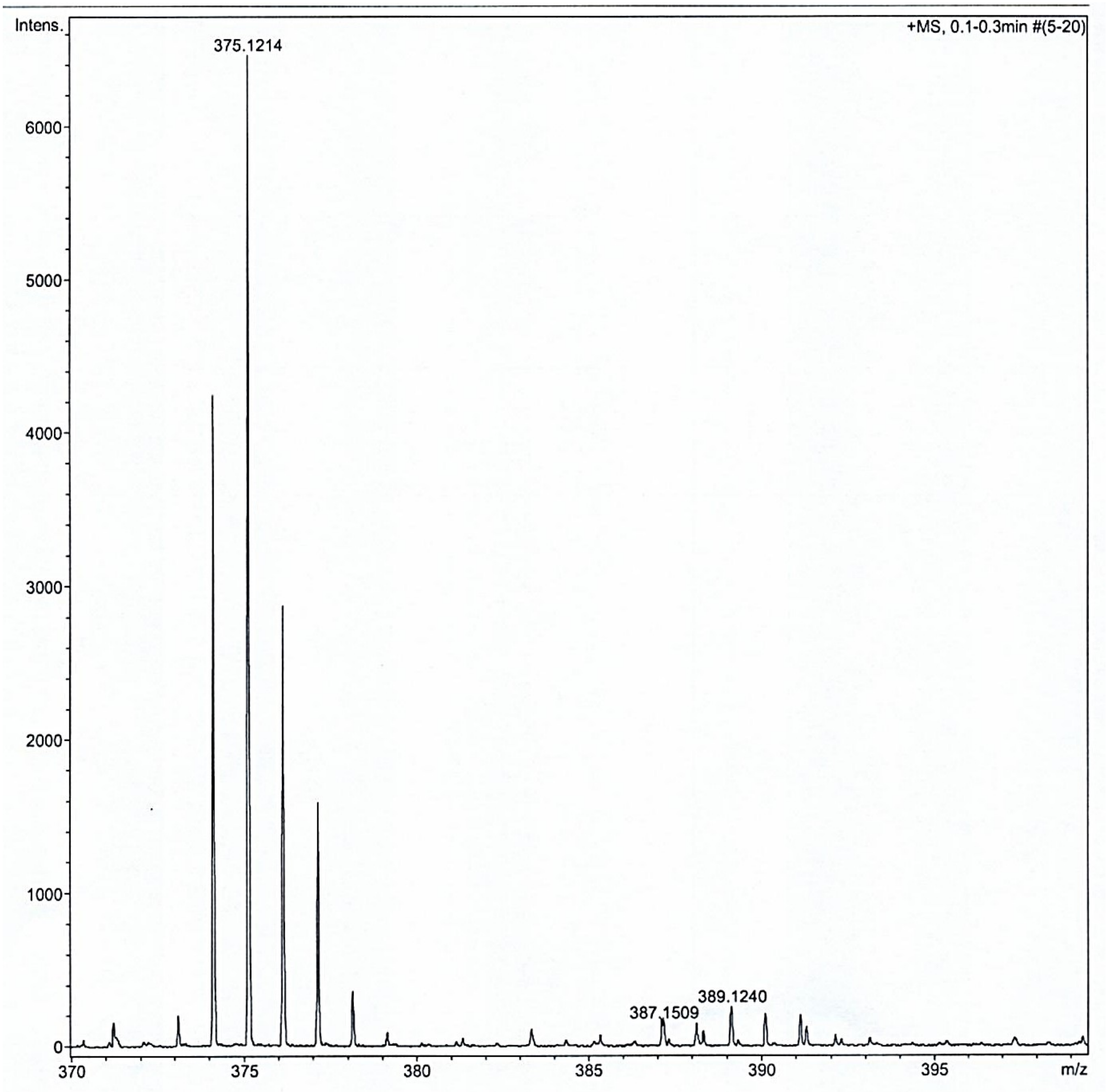

Figure 13S. APCI-MS spectrum of compound 3.

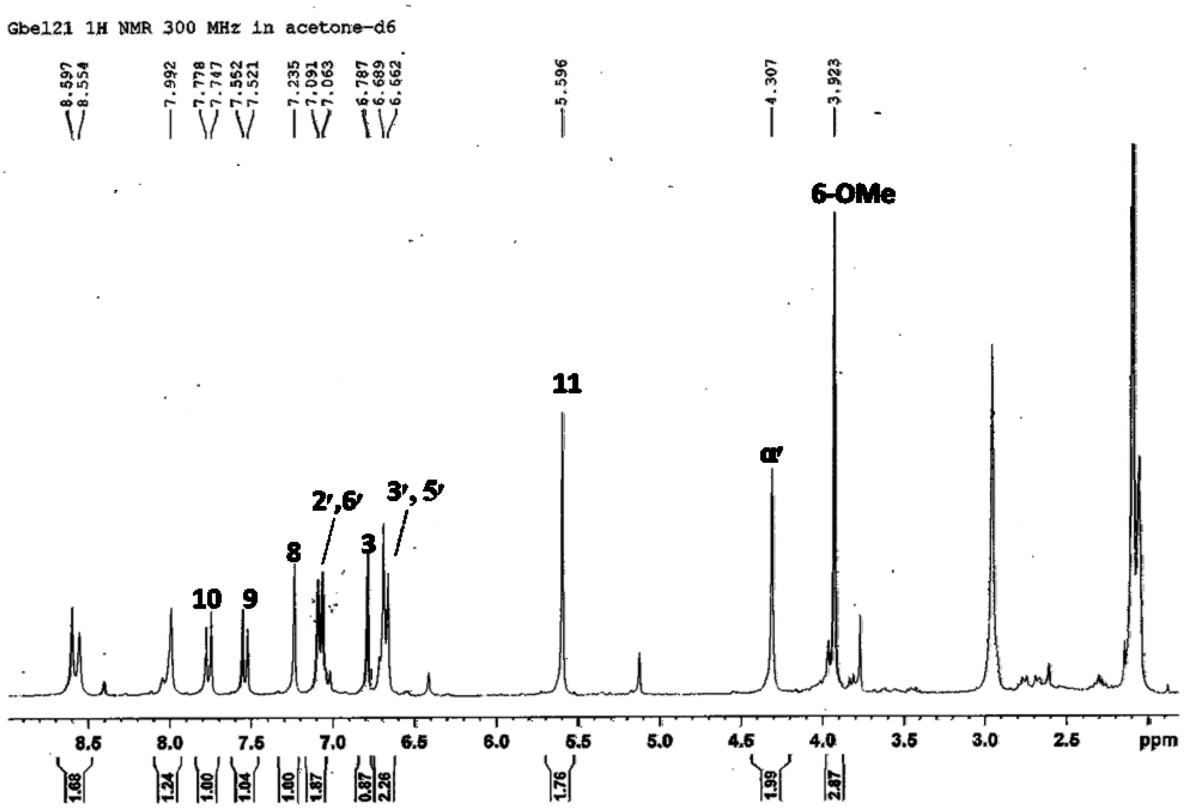

Figure 14S. ^{1}H NMR (acetone-d_6, 300 MHz) spectrum of compound 3.

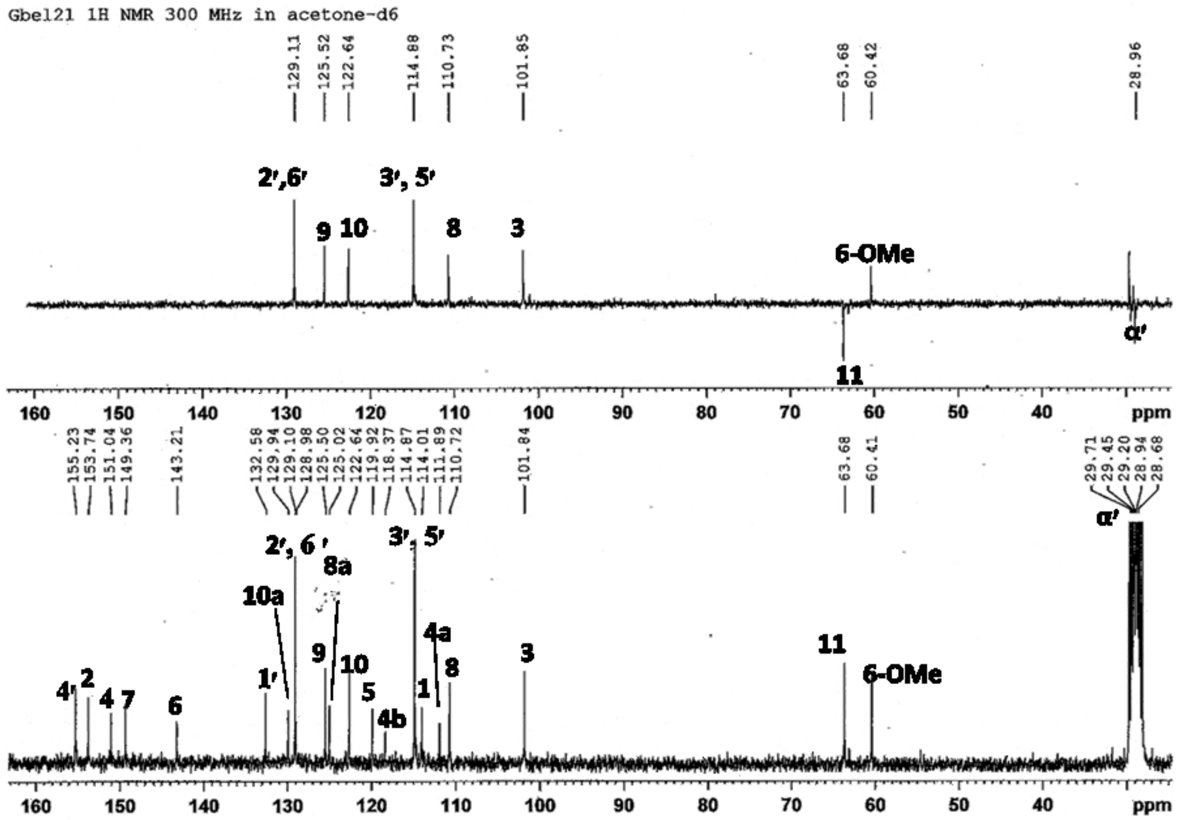

Figure 15S. ^{13}C NMR and DEPT (acetone-d_6, 75 MHz) spectrum of compound 3.

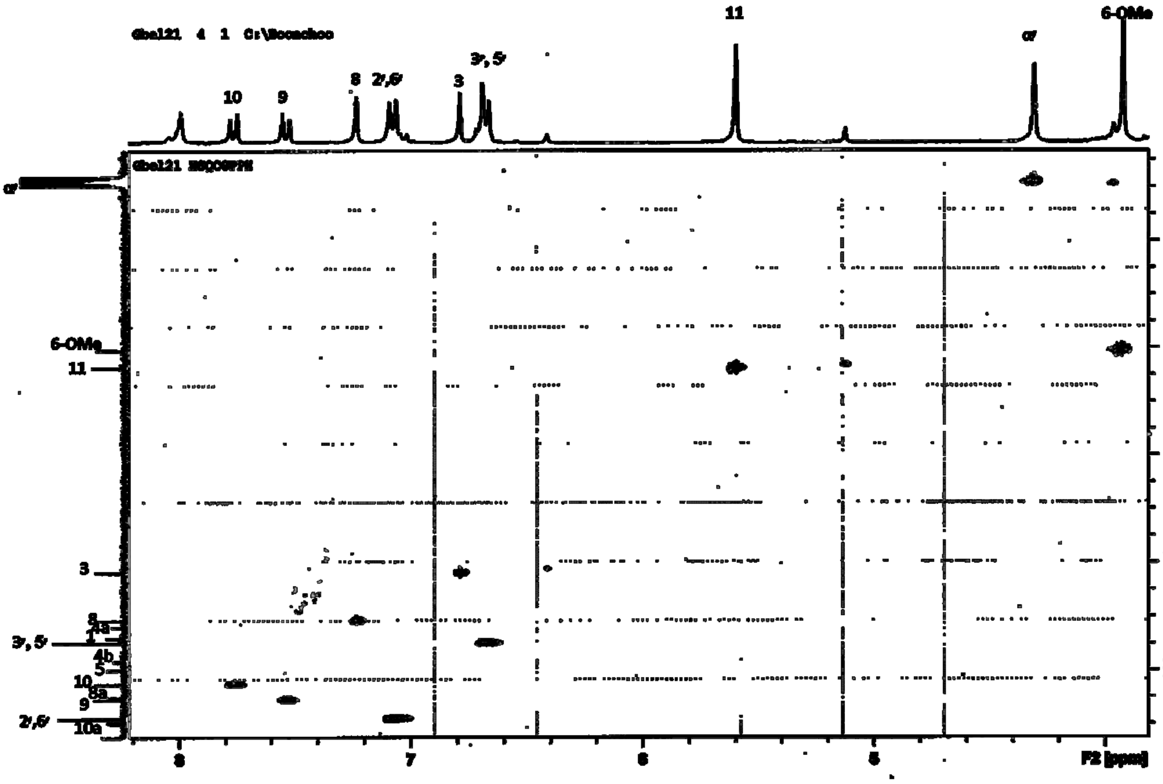

Figure 16S. HSQC (acetone-d_6, 300/75 MHz) spectrum of compound 3.

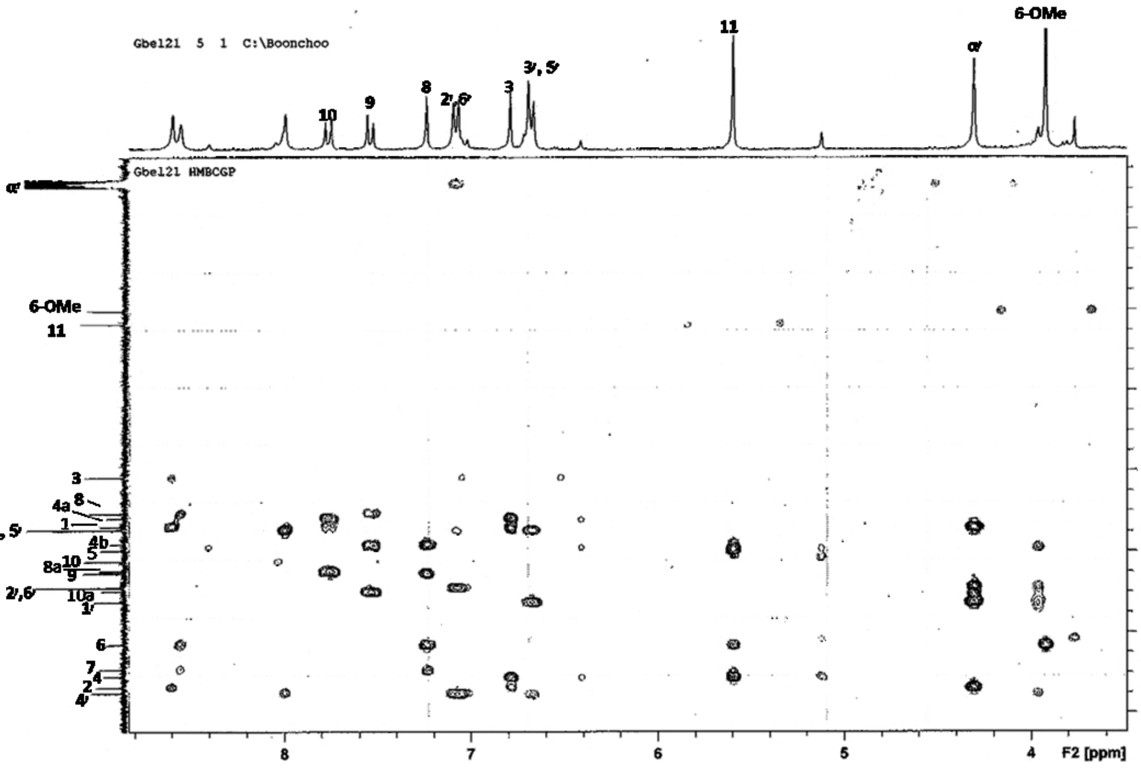

Figure 17S. HMBC (acetone-d_6, 300/75 MHz) spectrum of compound 3.

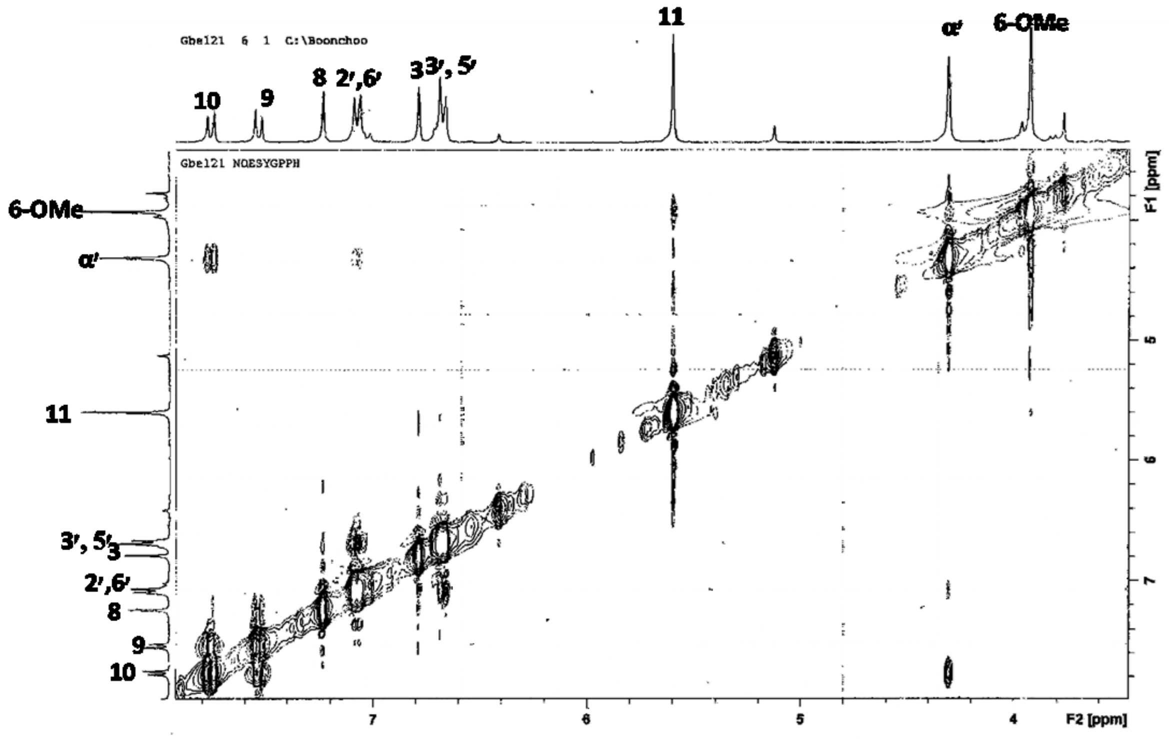

Figure 18S. NOESY (acetone-d_6, 300 MHz) spectrum of compound 3.

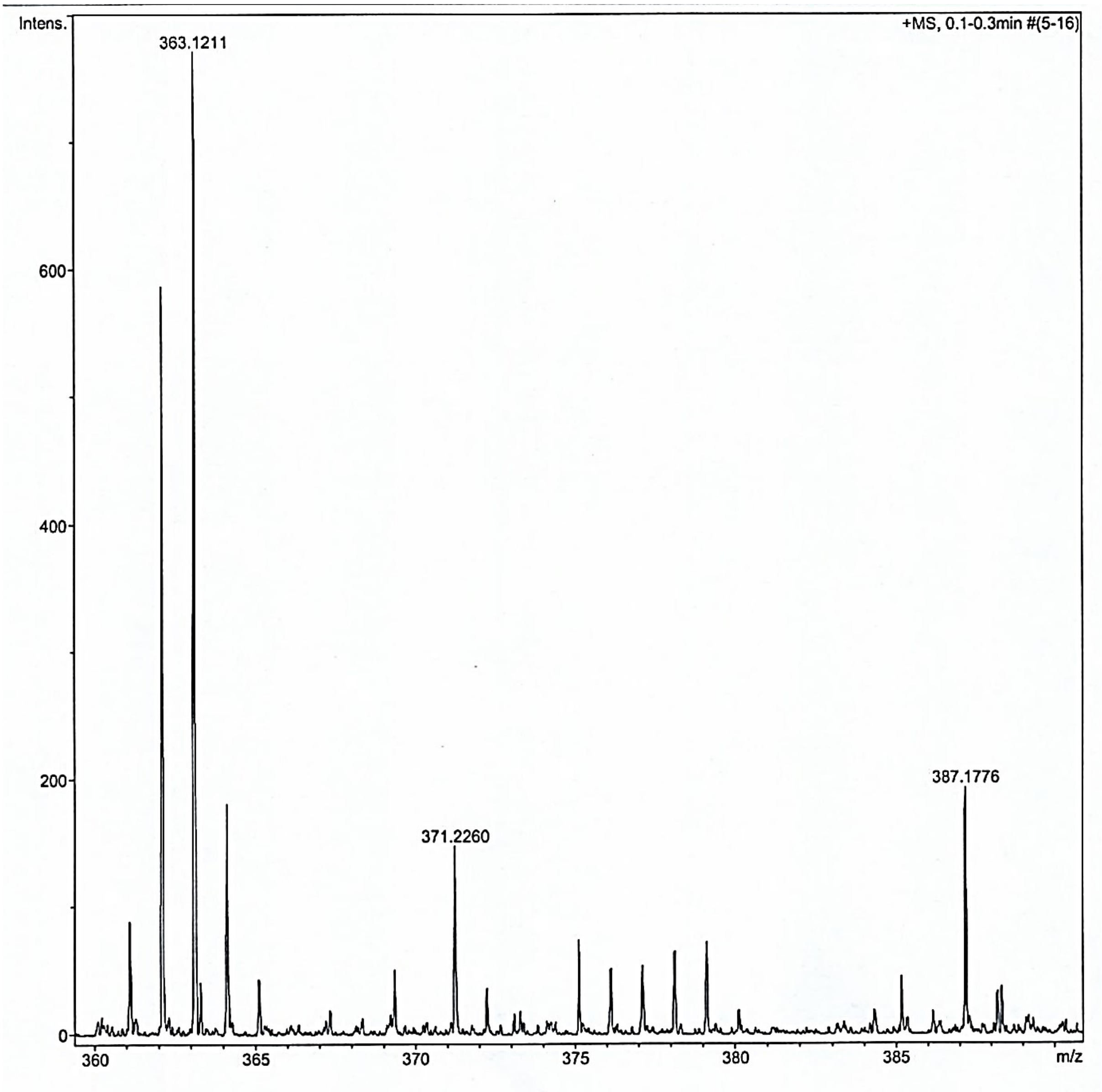

Figure 19S. APCI-MS spectrum of compound 4.

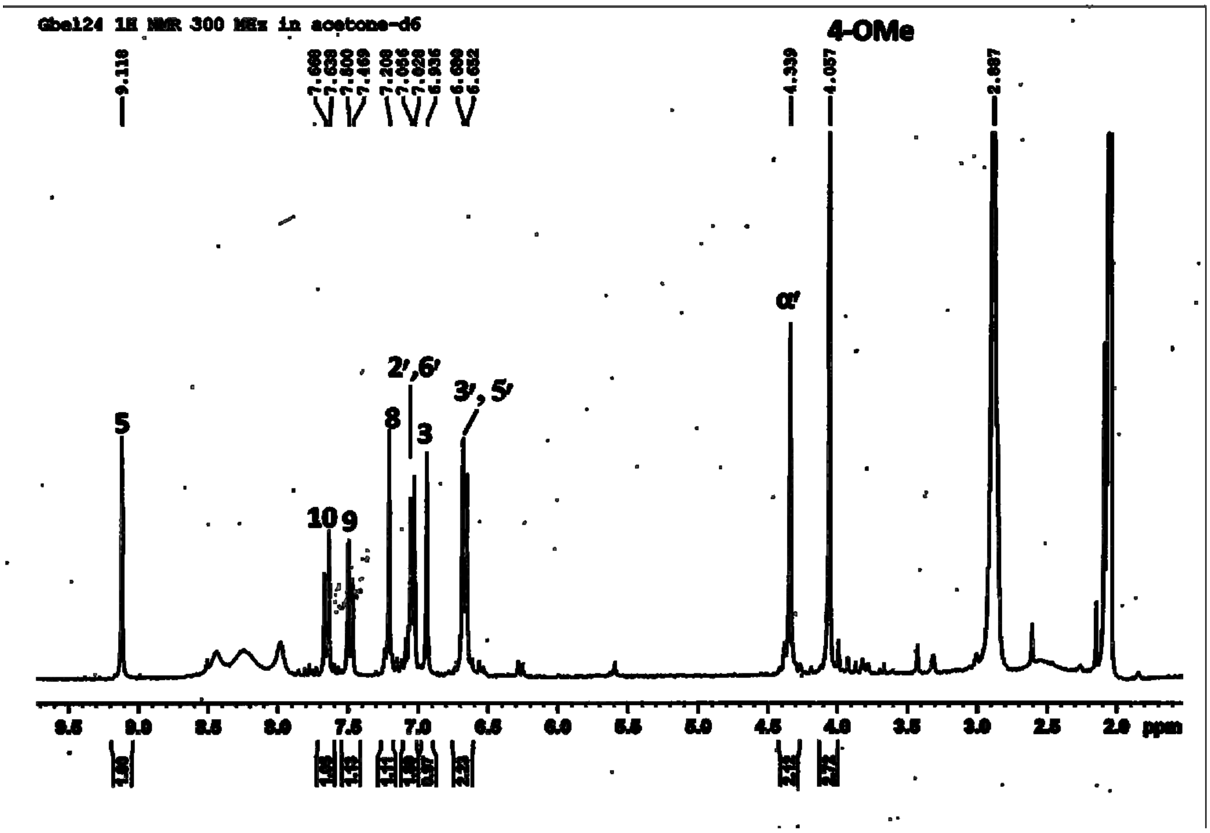

Figure 20S. ^{1}H NMR (acetone-d_6, 300 MHz) spectrum of compound 4.

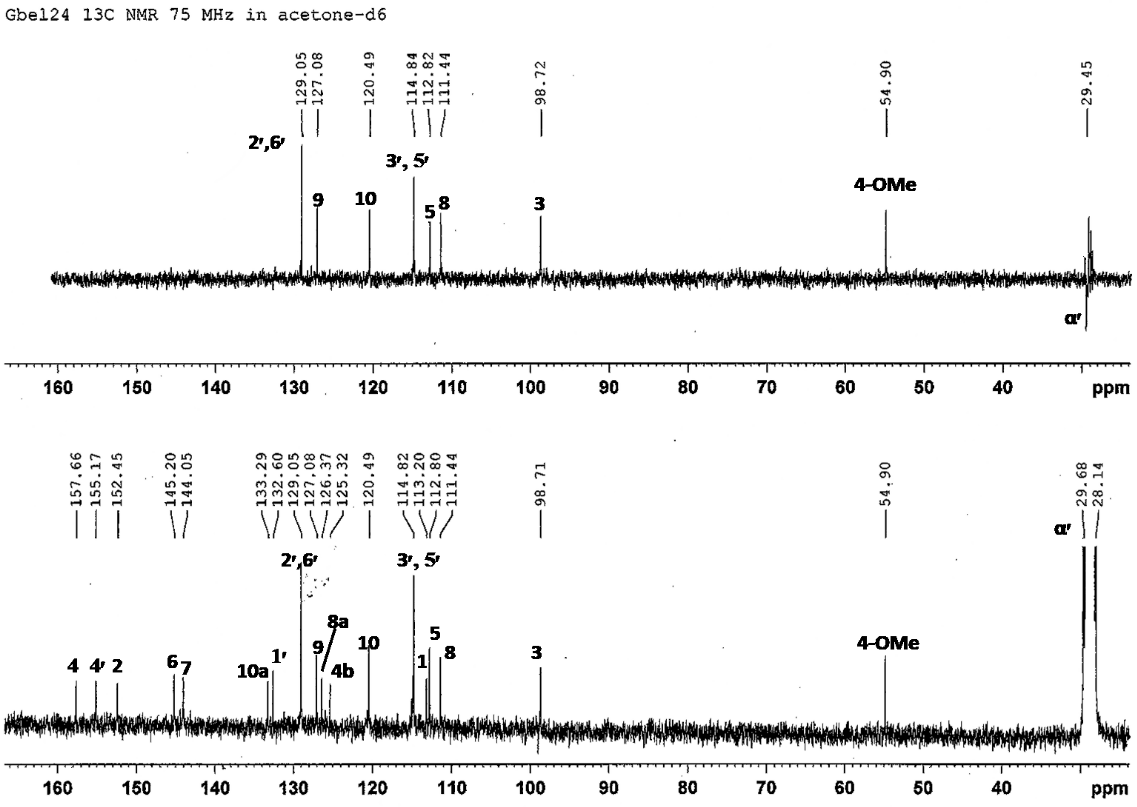

Figure 21S. ^{13}**C NMR and DEPT (acetone-d_6, 75 MHz) spectrum of compound 4.**

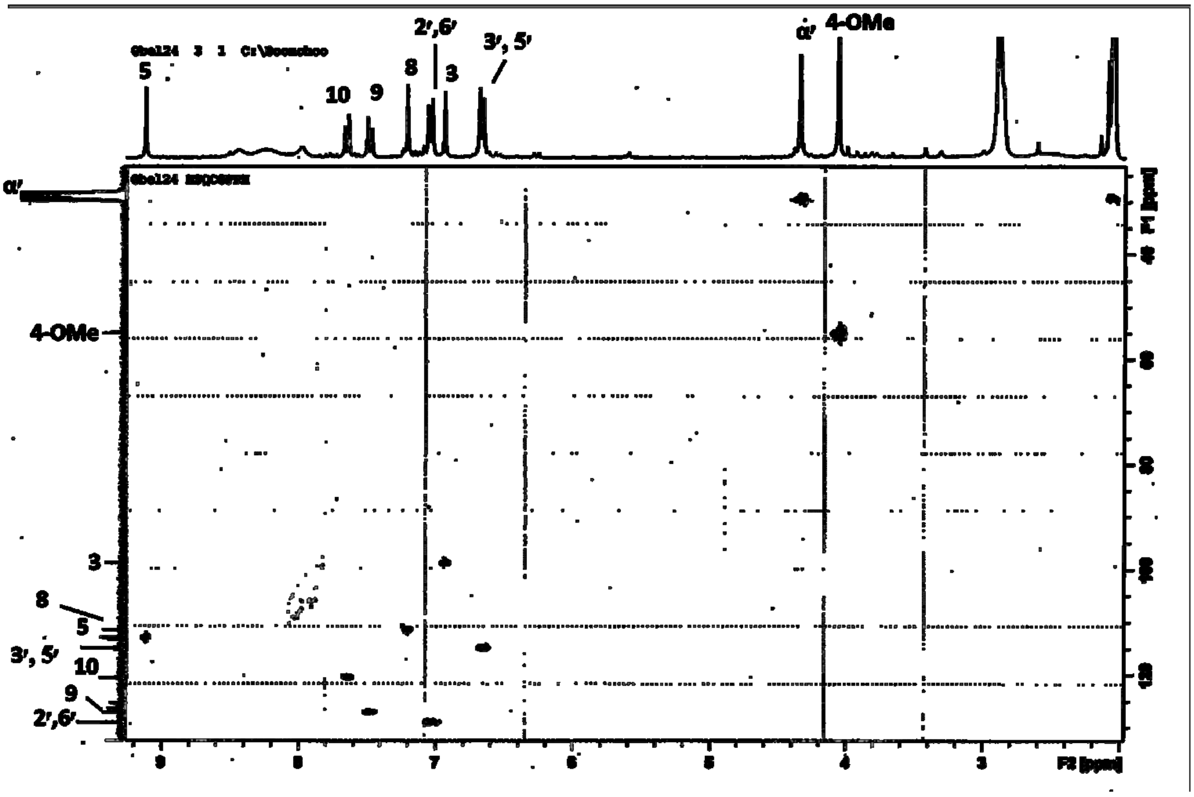

Figure 22S. HSQC (acetone-d_6, 300/75 MHz) spectrum of compound 4.

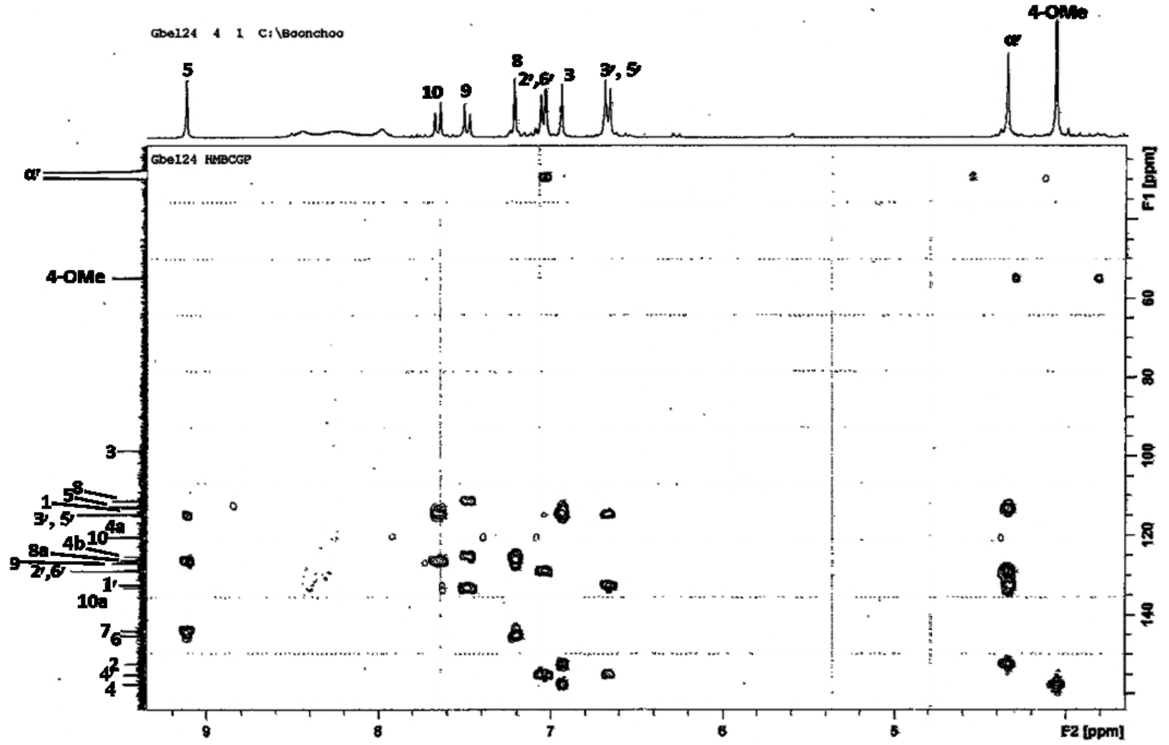

Figure 23S. HMBC (acetone-d_6, 300/75 MHz) spectrum of compound 4.

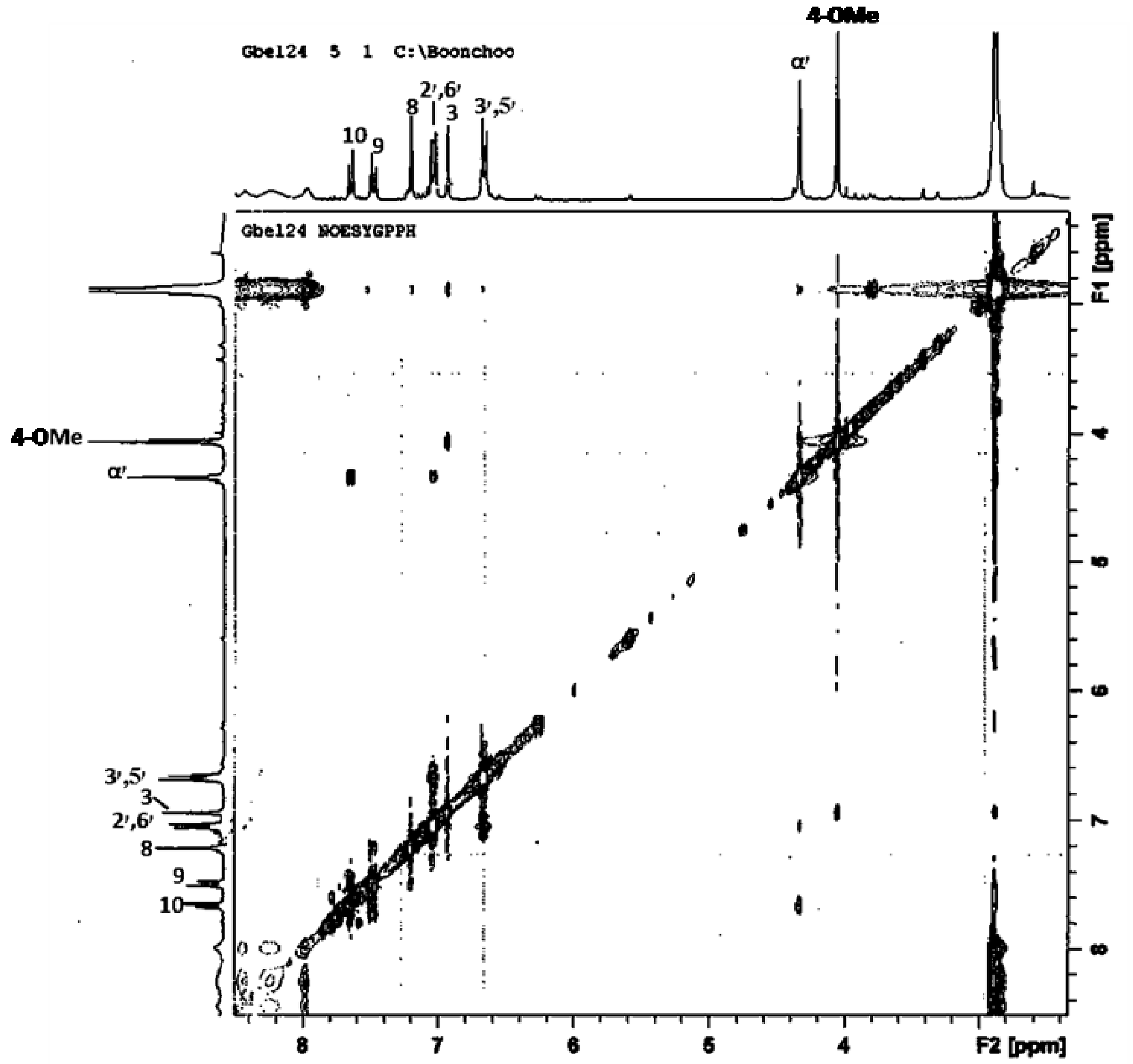

Figure 24S. NOESY (acetone-d_6, 300 MHz) spectrum of compound 4.
